# IL-33/ST2 induces neutrophil-dependent reactive oxygen species production and mediates gout pain

**DOI:** 10.7150/thno.48028

**Published:** 2020-10-27

**Authors:** Chengyu Yin, Boyu Liu, Yuanyuan Li, Xiaojie Li, Jie Wang, Ruixiang Chen, Yan Tai, Qiyang Shou, Ping Wang, Xiaomei Shao, Yi Liang, Hong Zhou, Wenli Mi, Jianqiao Fang, Boyi Liu

**Affiliations:** 1 Department of Neurobiology and Acupuncture Research, The Third Clinical Medical College, Zhejiang Chinese Medical University, Key Laboratory of Acupuncture and Neurology of Zhejiang Province, Hangzhou, 310053, China.; 2 Academy of Chinese Medical Sciences, Zhejiang Chinese Medical University, Hangzhou, 310053, China.; 3 The Second Clinical Medical College, Zhejiang Chinese Medical University, Hangzhou 310053, China.; 4 Department of Pathology, School of Basic Medical Science, Zhejiang Chinese Medical University, Hangzhou, 310053, China.; 5 Department of Immunology, Anhui Medical University, Hefei, 230032, China.; 6 Department of Integrative Medicine and Neurobiology, The Academy of Integrative Medicine, School of Basic Medical Sciences, Institutes of Brain Science, Brain Science Collaborative Innovation Center, State Key Laboratory of Medical Neurobiology, Fudan University, Shanghai, 200032, China.

**Keywords:** Gout, arthritis, TRPA1, reactive oxygen species, cytokine, neutrophil

## Abstract

**Objective:** Gout, induced by monosodium urate (MSU) crystal deposition in joint tissues, provokes severe pain and impacts life quality of patients. However, the mechanisms underlying gout pain are still incompletely understood.

**Methods:** We established a mouse gout model by intra-articularly injection of MSU crystals into the ankle joint of wild type and genetic knockout mice. RNA-Sequencing, *in vivo* molecular imaging, Ca^2+^ imaging, reactive oxygen species (ROS) generation, neutrophil influx and nocifensive behavioral assays, etc. were used.

**Results:** We found interleukin-33 (IL-33) was among the top up-regulated cytokines in the inflamed ankle. Neutralizing or genetic deletion of IL-33 or its receptor ST2 (suppression of tumorigenicity) significantly ameliorated pain hypersensitivities and inflammation. Mechanistically, IL-33 was largely released from infiltrated macrophages in inflamed ankle upon MSU stimulation. IL-33 promoted neutrophil influx and triggered neutrophil-dependent ROS production via ST2 during gout, which in turn, activated transient receptor potential ankyrin 1 (TRPA1) channel in dorsal root ganglion (DRG) neurons and produced nociception. Further, TRPA1 channel activity was significantly enhanced in DRG neurons that innervate the inflamed ankle via ST2 dependent mechanism, which results in exaggerated nociceptive response to endogenous ROS products during gout.

**Conclusions:** We demonstrated a previous unidentified role of IL-33/ST2 in mediating pain hypersensitivity and inflammation in a mouse gout model through promoting neutrophil-dependent ROS production and TRPA1 channel activation. Targeting IL-33/ST2 may represent a novel therapeutic approach to ameliorate gout pain and inflammation.

## Introduction

Gout is caused by monosodium urate (MSU) crystal accumulation in the joints and periarticular tissues 1. It is the most common inflammatory arthritis worldwide 1. Unfortunately, the incidence of gout is still increasing due to life style/dietary changes and the aging population 2. Patients with gout suffer from intense joint inflammation and excruciating arthritic pain, which severely reduced the patients' life quality 3. Gout attack is usually treated with colchicine, nonsteroidal anti-inflammatory drugs (NSAIDs) or corticosteroids 4-6. In spite of that, gout is still difficult to treat due to the many unwanted adverse effects of these conventional therapies 3. Currently, interleukin-1β (IL-1β) neutralizing monoclonal antibody and synthetic IL-1R antagonists are available alternative options 7, 8. But patients will have to be closely watched for intolerabilities or side effects 4, 9.

MSU provokes the activation of innate immune system and thereby, elicits strong inflammatory response in the joint and periarticular tissues 5, 10. Infiltrated macrophages engulf MSU via phagocytosis and subsequently release an array of inflammatory mediators 11. Some of these mediators may possibly interact with the peripheral nociceptors to elicit gout pain. In addition, our recent work, together with others, demonstrated that endogenous ROS products are critically involved in mediating gout pain and inflammation 12, 13. However, it still remains elusive how ROS is generated in the inflamed tissues in the context of gout and mediates gout pain.

It was recently reported that serum IL-33 expression was increased in gout patients compared to healthy controls and positively correlated with the inflammatory indicator C-reactive protein 14, 15. IL-33, a member of the IL-1 family, binds to the receptor complex, consisting of IL-33 specific ST2 and IL-1 receptor accessory protein IL-1RAcp, to elicit biological functions in inflammation, autoimmune response and homeostasis 16, 17. Recent evidence suggests that IL-33 can act as an important pain mediator in certain pain conditions, including joint pain, bone cancer pain, muscle pain and neuropathic pain 12, 18, 19. Our recent work further identified IL-33 engages with ST2 in peripheral sensory neurons to exert sensory neuron hyperexcitability 20, 21. These studies all demonstrate a pivotal role for IL-33/ST2 in mediating nociception and sensory neuron activities.

In the present study, we aimed to explore the potential pronociceptive or inflammatory mediators in the inflamed ankle from MSU-induced mouse gout arthritis model via RNA-Seq expression analysis. We further found that IL-33/ST2 mediates pain hypersensitivity and inflammation in the mouse gout model through promoting neutrophil-dependent ROS production and TRPA1 channel activation.

## Results

### MSU-induced gout arthritis triggers the release of IL-33 from the inflamed ankle tissues

**Figure 1:**
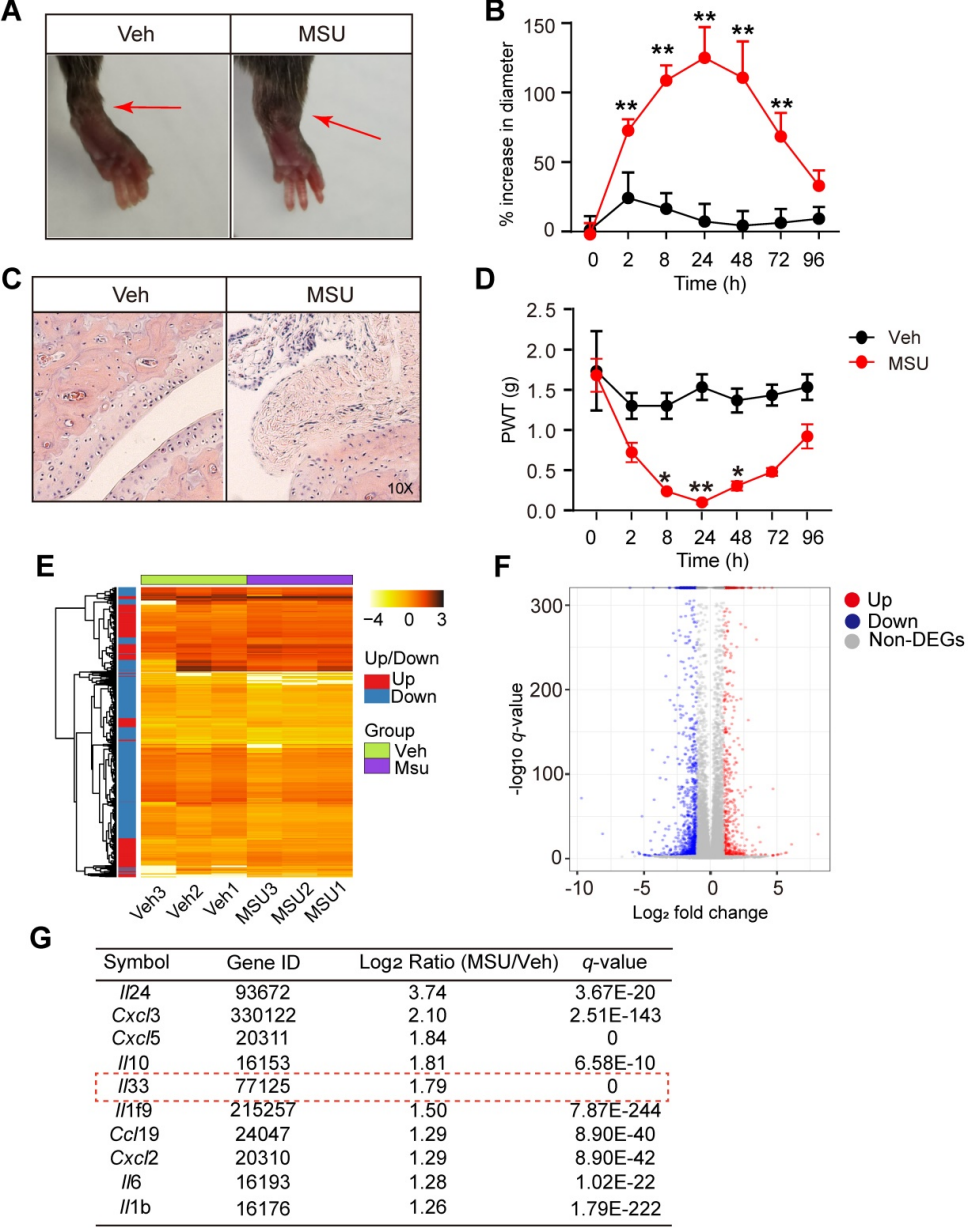
** RNA-Seq expression analysis of mRNA changes in inflamed ankle tissues of MSU-induced mouse gout model.** (A) Representative photos showing mouse ankle injected with PBS (Veh group) or MSU (MSU group). The pictures were taken 24 h after injection. (B) Time course of the % increase in ankle diameter after model establishment. n = 6 mice/group, ^**^p<0.01 *vs*. Veh group. (C) Representative photos of ankle H&E staining from Veh and MSU group. The right panels show enlarged area as indicated in the left. (D) Time course of PWT changes in the right hind paw after model establishment. n = 6 mice/group,^ *^p<0.05 and ^**^p<0.01 *vs*. Veh group. (E) Heat map illustration of the DEGs derived from MSU group *vs*. Veh group. n = 3 mice/group. (F) Volcano plot showing gene expression profiles in MSU group *vs*. Veh group. Red and blue spots indicate up- and down-regulated DEGs, respectively, whereas grey spots indicate non-DEGs. (G) Top 10 most highly up-regulated inflammatory cytokines or chemokines in MSU group *vs*. Veh group.

We first established the mouse model of gout arthritis by intra-articular (i.a.) injection of MSU (0.5 mg/20 μL) into mouse ankle (MSU group) as we previously described 12. Control animal received PBS injection (20 μl PBS, i.a., Veh group). MSU injection elicited obvious ankle edema, which showed up 2 h after injection and persisted until 72 h, compared with Veh group (Figure 1A & B). Pathology analysis indicated strong inflammatory cell infiltration into the ankle of MSU-treated mice (Figure 1C). Moreover, MSU-injected mice developed robust mechanical allodynia, manifested by reduction of paw withdraw threshold (PWT), which peaked at 24 h and lasted until 48 h (Figure 1D). To explore potential endogenous pain mediators in MSU-induced gout arthritis, we performed RNA-Seq to examine gene expression profiles in ankle tissues isolated from MSU-injected and vehicle-injected mice. Ankle tissues were collected 24 h after MSU or PBS injection and high quality RNA was extracted for RNA-Seq analysis (Figure S1A & B). A total of 1722 genes, which take up 8.9% of total genes (19323), was found to be significantly up- or down-regulated (DEGs, |log_2_ (Fold Change)| ≥1.0; q≤0.001) in ankle tissues of MSU-injected mice *vs*. vehicle-injected mice (Figure 1E). The 594 upregulated and 1128 downregulated DEGs were illustrated in scatter plot (Figure 1F).

Among these DEGs, we especially focused on inflammatory cytokines or chemokines that were upregulated. Figure 1G showed the top 10 inflammatory cytokines or chemokines identified to be significantly up-regulated. Among these genes, certain well-known inflammatory mediators, such as *Il-6*, *Il-1β* and* Cxcl3*, were significantly increased in MSU group. We were particular interested in *Il-33*, which has not previously been implicated in gout pain, but implicated in some other pain conditions, including neuropathic, cancer and muscle pain, etc. 18, 19 Therefore, we set to examine the possible involvement of IL-33 in mediating the pain response of the mouse gout model.

**Figure 2:**
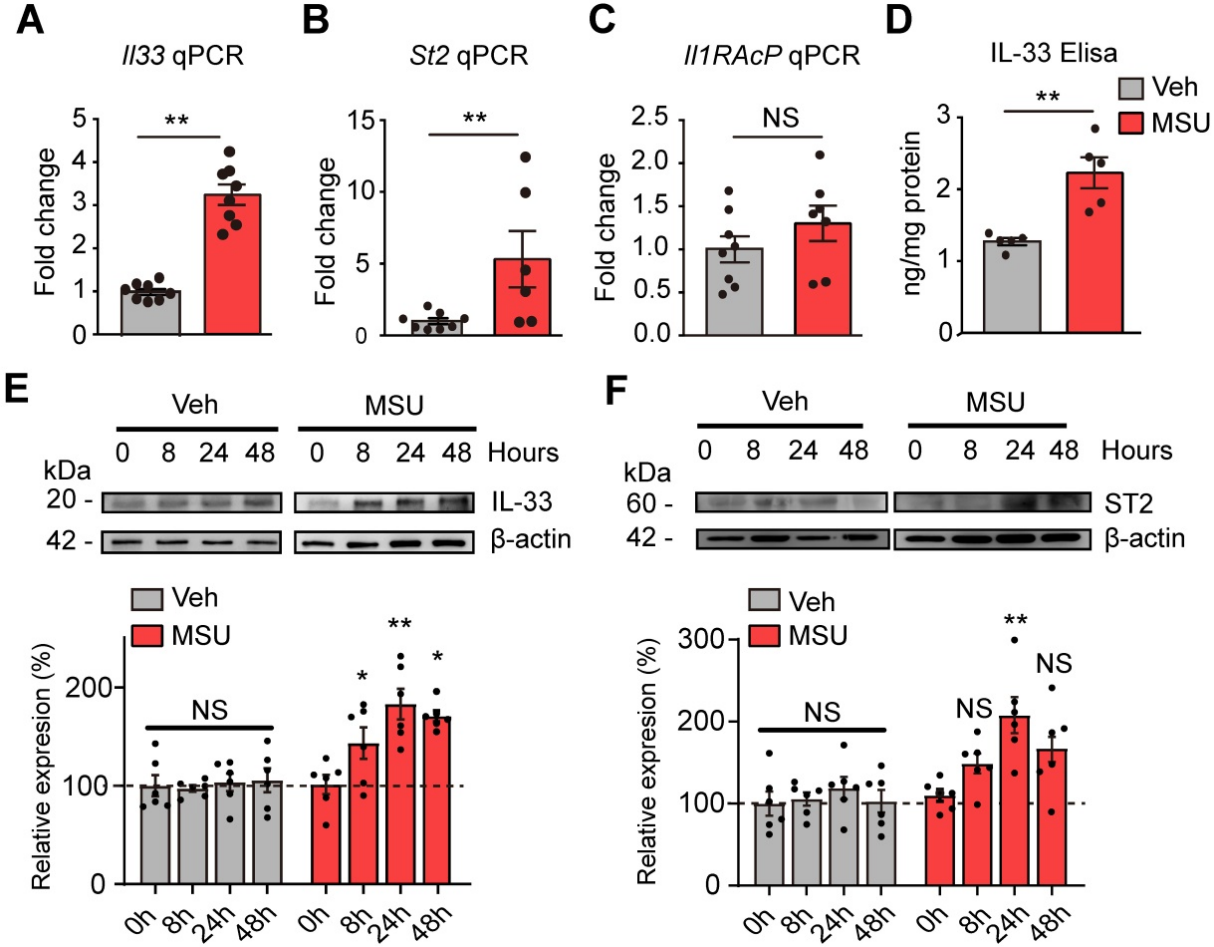
** The expressions of IL-33 and ST2 proteins are up-regulated in inflamed ankle tissues of mouse gout model.** (A-C) qPCR analysis of *Il-33*,* St2* and *Il-1RAcP* gene expression fold changes in MSU group *vs*. Veh group (PBS). Ankle tissues were harvested 24 h after model establishment. n = 6-8 mice/group. (D) ELISA result of IL-33 protein changes in MSU group *vs*. Veh group. n = 5 mice/group. ^**^p<0.01, NS: no significance. (E) IL-33 protein expression in ankle tissues examined by Western blotting at 0, 8, 24 and 48 h time points after MSU or Veh group establishment. Upper panel shows representative photos of IL-33 and β-actin protein expression. Lower panel indicates the summarized IL-33 expression normalized to β-actin in MSU and Veh group. n = 6 mice/group. (F) ST2 protein expression in ankle tissues examined by Western blotting at 0, 8, 24 and 48 h time points after MSU or Veh group establishment. Upper panel shows representative photos of ST2 and β-actin protein expression. Lower panel indicates the summarized ST2 expression normalized to β-actin in MSU and Veh group. n = 6 mice/group. ^*^p<0.05 and ^**^p<0.01 *vs*. 0 h time point, NS: no significance *vs.* 0 h time point. Student's* t* test was used for statistical analysis in (A-D). One-way ANOVA followed by Tukey post hoc test was used for statistical analysis in panels (E-F).

qPCR confirmed that *Il-33* gene were significantly increased in ankle tissues of MUS group (Figure 2A). *St2*, the gene encoding the specific receptor for IL-33, was significantly upregulated as well (Figure 2B). *Il-1RAcP*, which forms IL-33 receptor complex with ST2, was not changed (Figure 2C). ELISA confirmed a corresponding increase of IL-33 protein concentration in ankle tissues from MSU group mice 24 h after model establishment (Figure 2D). Western blot further indicated IL-33 protein expression was significantly increased in ankle tissues of MSU group 8, 24 and 48 h after model establishment, which peaked at 24 h time point (Figure 2E). ST2 protein expression in ankle tissue was also significantly increased 24 h after MSU injection (Figure 2F). In contrast, IL-33 or ST2 protein expression was not significantly changed in Veh group at all time points (Figure 2E & F).

### IL-33/ST2 signaling is involved in mediating the pain hypersensitivity and inflammatory response of the mouse gout model

**Figure 3:**
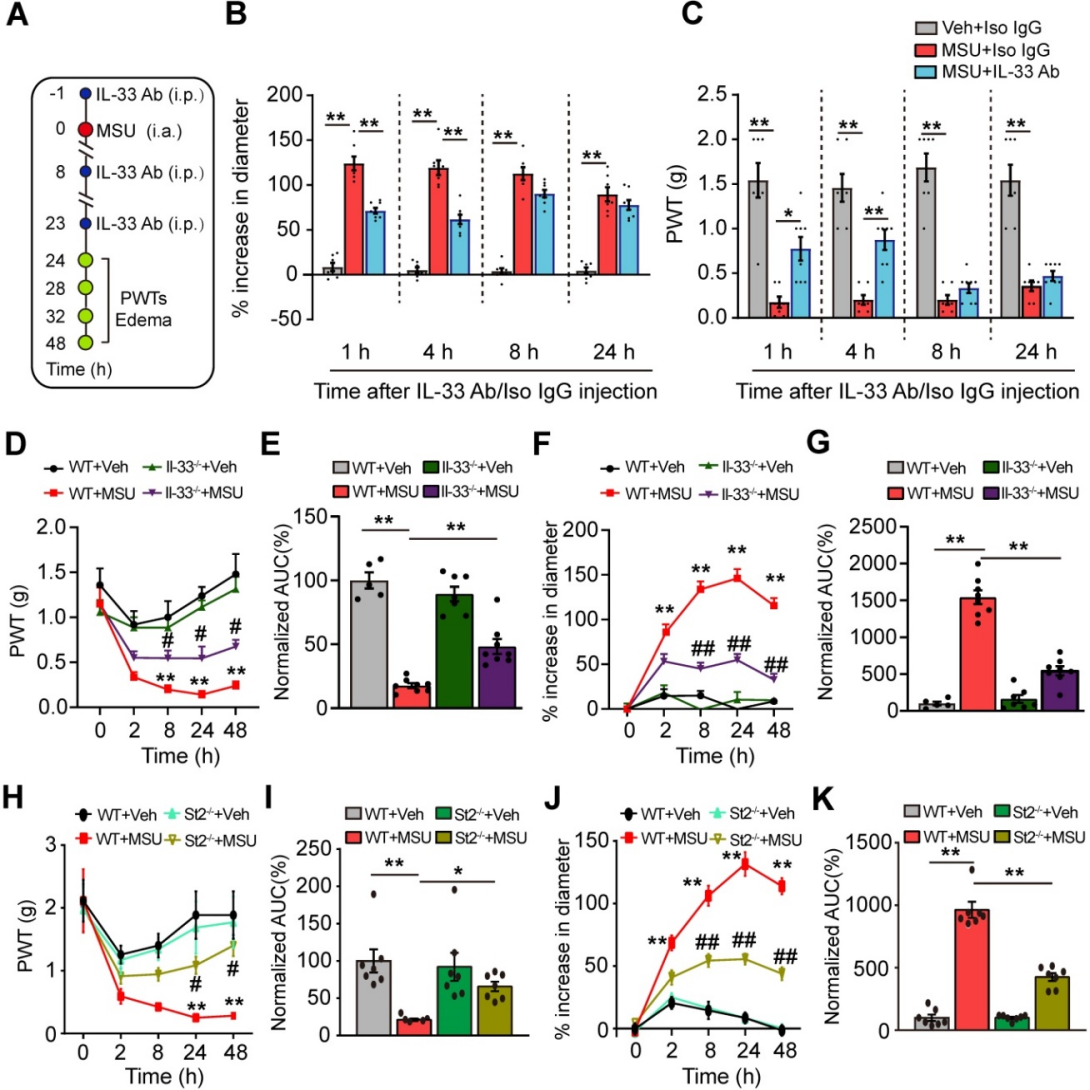
** IL-33/ST2 is involved in mediating pain hypersensitivity and inflammatory response of the mouse gout model.** (A) Schematic picture depicting the time points of IL-33 neutralizing antibody (IL-33 Ab) or isotype control IgG application (i.p.) and behavioral/ankle edema tests. MSU or PBS (Veh) was injected into the ankle (i.a.) at 0 h time point. (B&C) The comparison of % increase of ankle diameter (B) or PWT (C) 1, 4, 8 and 24 h after IL-33 Ab or isotype control IgG application. (D) Time course showing PWT changes in WT and Il33^-/-^ mice after MSU or PBS injection. (E) Summary of the normalized area under the curve as in (D), in which WT+Veh group was taken as 100%. n = 6 mice/group. (F) Time course showing % increase in ankle diameter in WT and Il33^-/-^ mice after MSU or PBS injection. (G) Summary of the normalized area under the curve as in (F), in which WT+Veh group was taken as 100%. n = 7 mice/group. (H) Time course showing PWT changes in WT and St2^-/-^ mice after MSU or PBS injection. (I) Summary of the normalized area under the curve as in (H), in which WT+Veh group was taken as 100%. n = 7 mice/group. (J) Time course showing % increase in ankle diameter in WT and St2^-/-^ mice after MSU or PBS injection. (K) Summary of the normalized area under the curve as in (J), in which WT+Veh group was taken as 100%. n = 7 mice/group. ^*^p<0.05 and ^**^p<0.01 *vs*. WT+Veh group. ^#^p<0.05 and ^##^p<0.01 *vs*. WT+MSU group. One-way ANOVA followed by Tukey post hoc test was used for statistical analysis in panels (E, G, I & K). Two-way ANOVA followed by Tukey post hoc test was used for statistical analysis in panels (B, C, D, F, H & J).

We set to study whether IL-33 was involved in mechanical allodynia of the mouse gout model. An IL-33 neutralizing antibody (5 μg/mouse, i.p.) or isotype control IgG (goat IgG, 5 μg/mouse, i.p.) was injected into mice 1 h before and 8, 23 h after model establishment (Figure 3A). IL-33 neutralizing antibody significantly reduced ankle edema and mechanical allodynia of MSU group mice at 24 and 28 h time points (Figure 3B & C). IL-33 neutralizing antibody gradually lost its effect 8 h after its last application (Figure 3B & C). We further examined the effects of IL-33 using *Il-33* deficient (Il-33^-/-^) mice. Similarly, Il-33^-/-^ mice exhibited reduced ankle edema and mechanical allodynia compared with wild type (WT) control group after model establishment (Figure 3D & F). Area under the curve (AUC) indicated accumulated reduction in mechanical allodynia and ankle edema in Il-33^-/-^+MSU group *vs*. WT+MSU group (Figure 3E & G). We then studied whether ST2 was also involved in the mechanical allodynia of mouse gout model using *St2* deficient (St2^-/-^) mice. When MSU was injected, St2^-/-^ mice (St2^-/-^+MSU) exhibited significantly reduced mechanical allodynia and ankle edema compared with WT (WT+MSU) group (Figure 3H & J). Area under the curve (AUC) indicated accumulated reduction in mechanical allodynia and ankle edema in St2^-/-^+MSU group compared with WT+MSU group (Figure 3I & K). Additionally, naïve Il-33^-/-^ and St2^-/-^ mice did not exhibit any deficits in general locomotor activities in open field test and body weight *vs*. WT mice (Figure S2A-D).

We then studied the behavioral effects of exogenously injecting IL-33 into gout model mice. 24 h after gout model establishment, recombinant mouse IL-33 (rIL-33) was injected into the ankle (3-300 ng/10 μl, i.a., Figure S3A) of the gout model mouse. 30 or 300 ng rIL-33 injection further enhanced the ankle edema and exacerbated the mechanical allodynia of the gouty mice at both 24.75 and 28 h time points compared with vehicle (BSA)-injected gouty mice, whereas 3 ng rIL-33 was not effective (Figure S3B & C). We further tested whether IL-33's effect is mediated via ST2. We found that co-injection of ST2 neutralizing antibody with rIL-33 largely abolished rIL-33's effect on ankle edema and 50% PWTs in MSU-treated WT mice (Figure S3D & E). Furthermore, rIL-33 injection (300 ng) into MSU-treated St2^-/-^ mice did not further induce ankle edema and mechanical allodynia compared with BSA (vehicle) (Figure S3F), which is consistent with the results derived from ST2 neutralizing antibody. Therefore, these data suggest that exogenous IL-33 acts on ST2 to exacerbate inflammation and pain hypersensitivities in gout model mice.

We further examined the expression of some key inflammatory cytokines and chemokines in ankle tissues via Bio-Plex multiplex immunoassays. As shown in Table 1, MSU treatment triggered upregulation of a number of cytokines and chemokines in ankle tissues of WT mice. Compared with WT mice, St2^-/-^ mice showed significantly reduced expression of IL-1β, IL-6, IL-13, CCL11, G-CSF, CXCL1, CCL2 and CCL3 proteins in ankle tissues (Table 1), suggesting St2^-/-^ mice showed generally attenuated inflammatory response during gout.

### Macrophage is among one of the cellular mechanisms for IL-33 overproduction in gout condition

**Figure 4:**
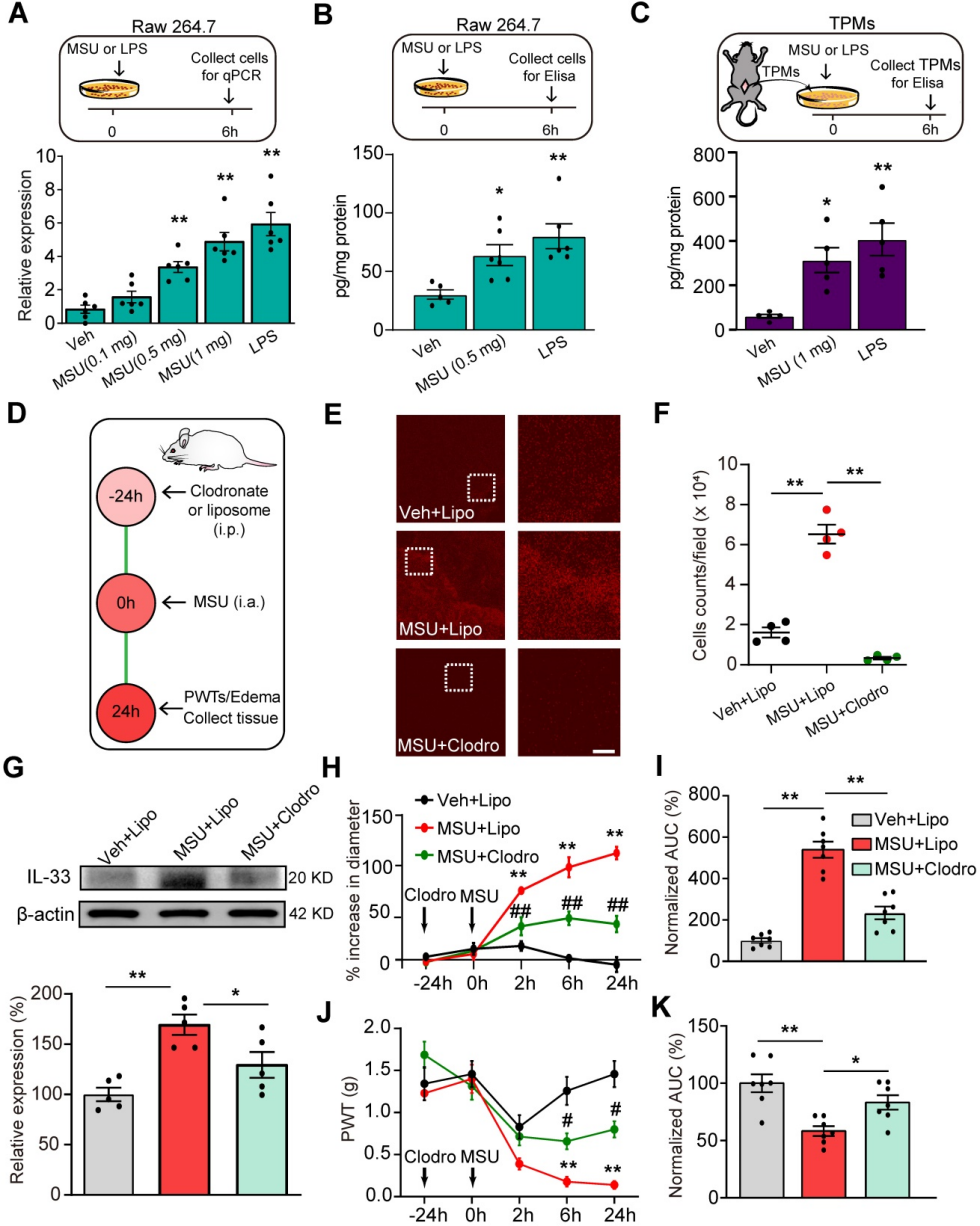
** Macrophage is a cellular mechanism for IL-33 overproduction during gout. (A)** Upper panel: Schematic protocol illustrating time points for MSU/LPS incubation and RAW264.7 cell harvest. Lower panel: Expression of *Il-33* gene in RAW264.7 cells after vehicle (Veh, PBS), MSU (0.5 or 1 mg/ml) or LPS (1 µg/ml) incubation by qPCR. (B) IL-33 protein expression changes in RAW264.7 cells after vehicle (PBS), MSU (0.5 mg/ml) or LPS (1 µg/ml) incubation by ELISA. (C) IL-33 protein expression changes in macrophages after vehicle, MSU (0.5 or 1 mg/ml) or LPS (1 µg/ml) incubation by ELISA. n = 5-6 replicates/group. (D) Protocol illustrating *in vivo* depleting macrophages in mice via treating with clodronate-laden liposome (i.p.). Liposome (Lipo) was used as vehicle control. (E) Immunofluorescence staining of periarticular tissues using F4/80 to detect macrophages. Right panels show enlarged fields depicted as white boxes on the left. (F) F4/80 positively stained cell counts per observation field. n = 4 mice/group. (G) Western blot of IL-33 protein expression in ankle tissues after macrophage depletion. Upper panel: representative images of IL-33 and β-actin protein expression in Veh+Lipo, MSU+Lipo and MSU+Clodro groups. Lower panel: summarized IL-33 expression normalized to β-actin. (H) Time course of the % increase in ankle diameter after MSU injection. (I) Summary of the normalized AUC as in (H), in which Veh+Lipo group was taken as 100%. (J) Time course of PWT changes after MSU injection. (K) Summary of the normalized AUC as in (J), in which Veh+Lipo group was taken as 100%. n = 7 mice/group. ^*^p<0.05 and ^**^p<0.01 *vs*. Veh+Lipo group. ^#^p<0.05 and ^##^p<0.01 *vs*. MSU+Lipo group. NS: no significance. One-way ANOVA followed by Tukey post hoc test was used for statistical analysis in panels (A, B, C, F, G, I & K). Two-way ANOVA followed by Tukey post hoc test was used for statistical analysis in panels (H & J).

IL-33 expression can be induced in macrophages by the bacterial endotoxin, lipopolysaccharide (LPS) 22-24. As macrophages accumulate in the inflamed tissues to react to MSU deposition by uptake of the crystals via phagocytosis 7, we set to test whether MSU could trigger IL-33 expression in macrophages. We first tested the effect of MSU on macrophage cell line RAW264.7 *in vitro*. As a positive control, incubation with LPS (1 μg/ml) induced obvious upregulation of *Il-33* gene expression in RAW264.7 cells compared with vehicle (PBS) incubated cells (Figure 4A), a result consistent with previous studies 22. MSU (0.5 or 1.0 mg/ml) incubation triggered a significant upregulation of *Il-33* gene expression in RAW264.7 cells (Figure 4A). ELISA test further confirmed that MSU incubation promoted IL-33 protein expression in RAW264.7 cells (Figure 4B). We then tested the effect of MSU on mouse macrophages. Thioglycollate-elicited macrophages (TPMs) were harvested from mice and exhibited over 90% macrophages purity examined by flow cytometry (Figure S4A & B). MSU (1 mg/ml) or LPS incubation (1 μg/ml) resulted in a significant increase in IL-33 protein expression in TMPs (Figure 4C).

We further examined the effect of *in vivo* macrophages depletion using clodronate-containing liposomes on IL-33 release in gout (Figure 4D). Clodronate (1 mg/100 μl, i.p.) robustly reduced the macrophages detected by F4/80 staining in the spleen of naïve mice compared with vehicle (liposome) treatment (Figure S5A & B), demonstrating the effectiveness of clodronate on macrophage depletion. MSU-treated mice showed obvious macrophage accumulation in periarticular ankle tissues, which was significantly reduced by clodronate treatment (Figure 4E & F). The ankle tissues were then collected and subjected to IL-33 protein detection. Clodronate-induced macrophage depletion significantly reduced the upregulation of IL-33 protein expression in ankle tissues from MSU-treated mice (Figure 4G). Clodronate-induced macrophage depletion further reduced ankle edema and mechanical allodynia of MSU-treated mice (Figure 4H-K). Based upon the above data, we reasoned that macrophage might be among one of the cellular mechanisms for IL-33 overproduction during gout.

### IL-33/ST2 signaling participates in oxidative stress modulation and promotes ROS production in gout

**Figure 5:**
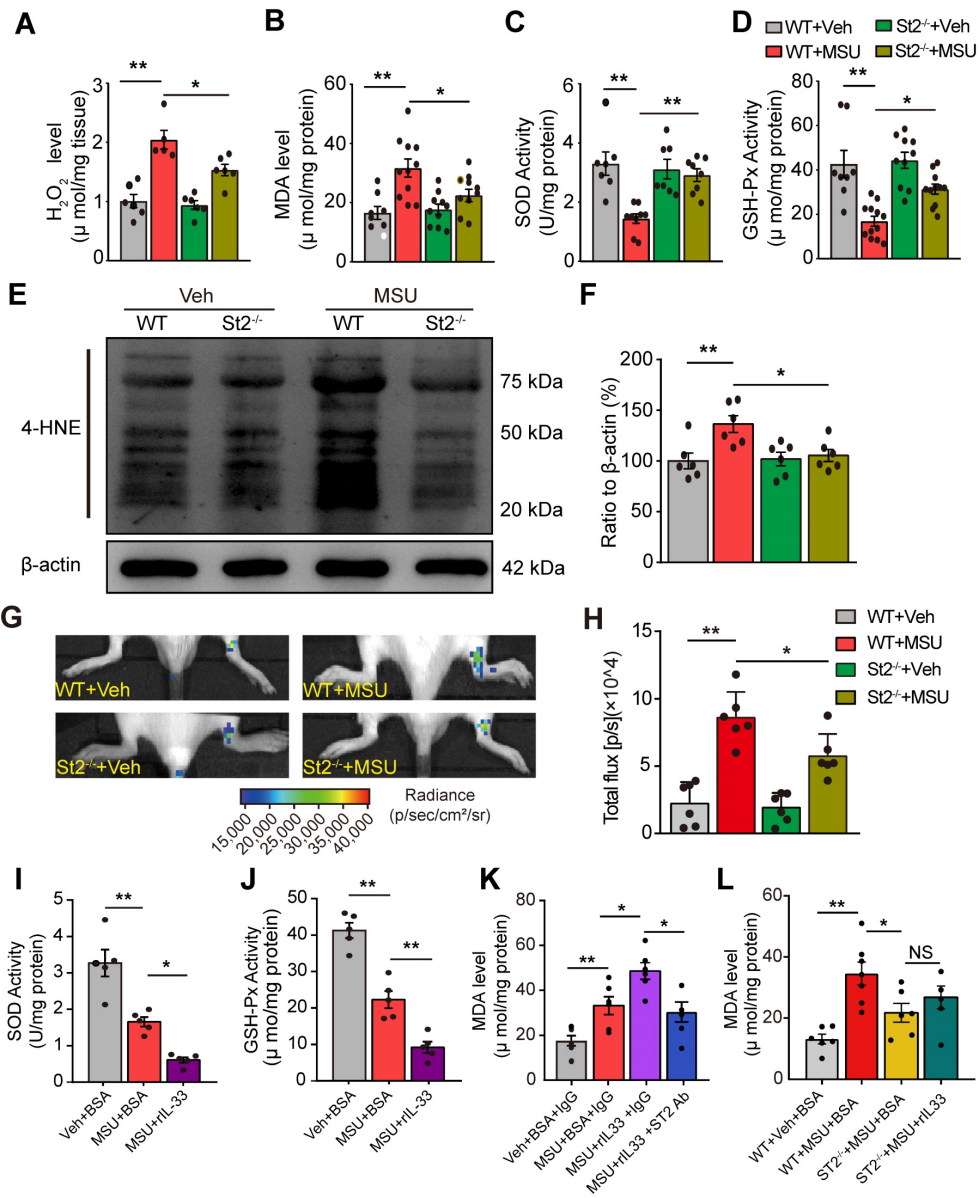
** IL-33/ST2 participates in oxidative stress modulation and promotes ROS production in gout condition.** (A-D) Summarized data showing H_2_O_2_ content (A), MDA content (B), SOD activity (C) and GSH-Px activity (D) determined in WT or ST2^-/-^ mice ankle tissues 24 h after vehicle (Veh, PBS) or MSU injection. (E) 4-HNE and β-actin expression in mice ankle tissues determined by Western blot. (F) Summarized data of 4-HNE expression normalized to β-actin. (G) *In vivo* ROS imaging showing ROS contents in the ankle area using L-012 in different groups. (H) Summarized total fluorescence flux of L-012 in the ankle area. (I-J) SOD activity (I) and GSH-Px activity (J) in ankle tissues 4 h after BSA or rIL-33 (300 ng) injection. (K) MDA level in ankle tissues 4 h after BSA or rIL-33 injection. ST2 neutralizing antibody (50 µg) or isotype IgG was co-injected with rIL-33. (L) MDA level in ankle tissues 4 h after BSA or rIL-33 injection in WT or St2^-/-^ mice. ^*^p<0.05 and ^**^p<0.01. NS: no significance. One-way ANOVA followed by Tukey post hoc test was used for statistical analysis.

We next explored the mechanisms underlying how IL-33/ST2 mediates the pain response of mouse gout model. We and others have demonstrated that endogenous ROS products resulted from oxidative stress played an important role in mediating gout pain and inflammation 12, 13, 25. Therefore, we began to explore whether IL-33/ST2 could have any effect on endogenous ROS production and oxidative stress level under gout condition. We found that H_2_O_2_ level was significantly increased in ankle tissues from WT+MSU group mice *vs*. WT+Veh group mice, whereas St2^-/-^+MSU group mice showed significantly reduced H_2_O_2_ level *vs*. WT+MSU group mice (Figure 5A). Furthermore, the levels of some lipid perioxidation products, such as malondialdehyde (MDA) and 4-hydroxynonenal (4-HNE), were both significantly increased in ankle tissues from WT+MSU group mice *vs*. WT+Veh group mice. In comparison, St2^-/-^+MSU group mice showed significantly reduced levels of MDA and 4-HNE (Figure 5B, E & F). We also evaluated the activities of antioxidant enzymes superoxidase dismutase (SOD) and glutathione peroxidase (GSH-Px). The levels of SOD and GSH-Px were dramatically decreased in ankle tissues of WT+MSU group mice, whereas St2^-/-^+MSU group mice showed significantly improved levels compared with WT+MSU group (Figure 5C&D). We further performed a noninvasive *in vivo* imaging of ROS productions in mouse ankle using the chemiluminescent probe L-012 26. WT+MSU group displayed significantly increased L-012 chemiluminescent signals in the ankle compared with MSU+Veh group, a sign of increased ROS production* in vivo*. In contrast, St2^-/-^+MSU group showed significantly reduced L-012 chemiluminescent signals *vs*. WT+MSU group (Figure 5G & H).

We then tested the effect of exogenously applied rIL-33 on oxidative stress in ankle tissues of the mouse gout model. Injection of rIL-33 (300 ng/mouse) into MSU-treated mice (MSU+rIL-33 group) promoted a further reduction of antioxidant enzymes SOD and GSH-Px activities and a further increase in MDA level in ankle tissues *vs*. MSU-treated mice injected with BSA (MSU+BSA group, Figure 5I, J & K). Furthermore, rIL-33-induced MDA level increase in ankle tissues of gout model mice was abrogated by co-administration with ST2 neutralizing antibody or in St2^-/-^ mice (Figure 5K & L). Collectively, the above results demonstrated that IL-33/ST2 promotes oxidative stress and ROS production in gout.

### IL-33/ST2 signaling drives neutrophil influx to produce oxidative stress in gout condition

**Figure 6:**
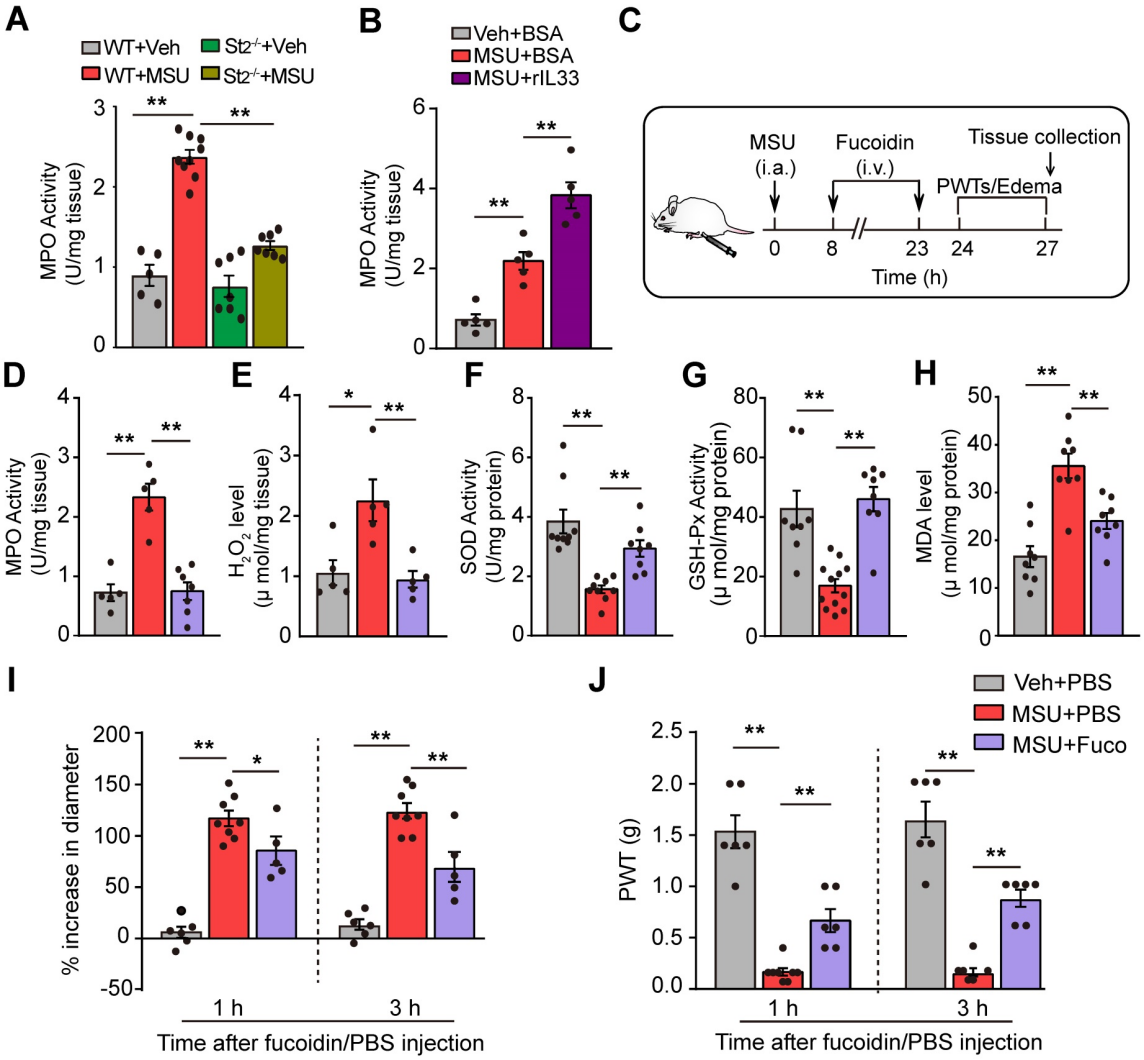
** IL-33/ST2 promotes neutrophil influx into inflamed tissue to generate oxidative stress in gout condition.** (A) MPO activity determined in WT or ST2^-/-^ mice ankle tissues 24 h after vehicle (Veh, PBS) or MSU injection. n = 5-9 mice/group. (B) MPO activity determined in ankle tissue of gout model mice after exogenously injection with BSA or rIL-33. (C) Schematic protocol for blocking neutrophil influx via fucoidine (20 mg/kg in 20 µl injecting volume, i.v.). (D-H) MPO activity (D), H_2_O_2_ (E), SOD activity (F), GSH-Px activity (G) and MDA content (H) measured in fucoidine- or vehicle (PBS)-treated gout model mice. n = 5-9 mice/group. (I) % increase in ankle diameter of gout model mice 1 or 3 h after fucoidine or PBS treatment. (J) PWT of gout model mice 1 or 3 h after fucoidine or PBS treatment. n = 5-8 mice/group. ^*^p<0.05 and ^**^p<0.01. One-way ANOVA followed by Tukey post hoc test was used for statistical analysis.

We then explored the mechanisms underlying how IL-33/ST2 promotes oxidative stress and ROS production. Neutrophils played an important role in mediating gout pain 27. Neutrophil exerts respiratory burst that rapidly releases ROS products 28-30. We found that myeloperoxidase (MPO) activity, a quantitative measurement of neutrophil congregation 27, was significantly increased in the inflamed ankles of MSU group mice, whereas MPO activity was significantly attenuated in St2^-/-^ mice (Figure 6A). Besides, injecting rIL-33 into the inflamed ankles of MSU group mice resulted in further increase in MPO activity (Figure 6B). We further established the mouse MSU air pouch gout model, which allows for convenient collection of adequate tissue exudates for cell infiltration analysis. Cytopathological analysis indicated that MSU injection into air pouches elicited an increase in the numbers of total cells, including neutrophils, lymphocytes and monocytes, among which neutrophils are the predominant cell type infiltrated. The numbers of total cells and neutrophils were both largely reduced in St2^-/-^ mice (Figure S7).

We continued to examine whether neutrophil was the cellular source for oxidative stress and ROS production in gout condition. We used fucoidin, a potent selectin inhibitor that reduces the aggregation of neutrophils, to reduce neutrophil influx 27. Fucoidin (20 mg/kg) was applied intravenously twice into MSU-treated mice (Figure 6C). Fucoidin treatment almost completely inhibited neutrophil influx into the ankle tissue after MSU injection, as indicated by a change in MPO activity (Figure 6D). Moreover, fucoidin significantly reduced ROS production and improved antioxidant enzyme activities in ankle tissues of MSU-treated mice (Figure 6E-H). Fucoidin treatment also attenuated the ankle edema and mechanical allodynia of MSU-treated mice (Figure 6I & J). These data suggest that IL-33/ST2 drives neutrophil influx and triggers neutrophil-dependent ROS production, resulting in mechanical allodynia of gout model mice.

### TRPA1 channel activity is increased in DRG neurons of gout model mice via ST2 dependent mechanism, resulting in enhanced nociceptive response to endogenous ROS products

**Figure 7:**
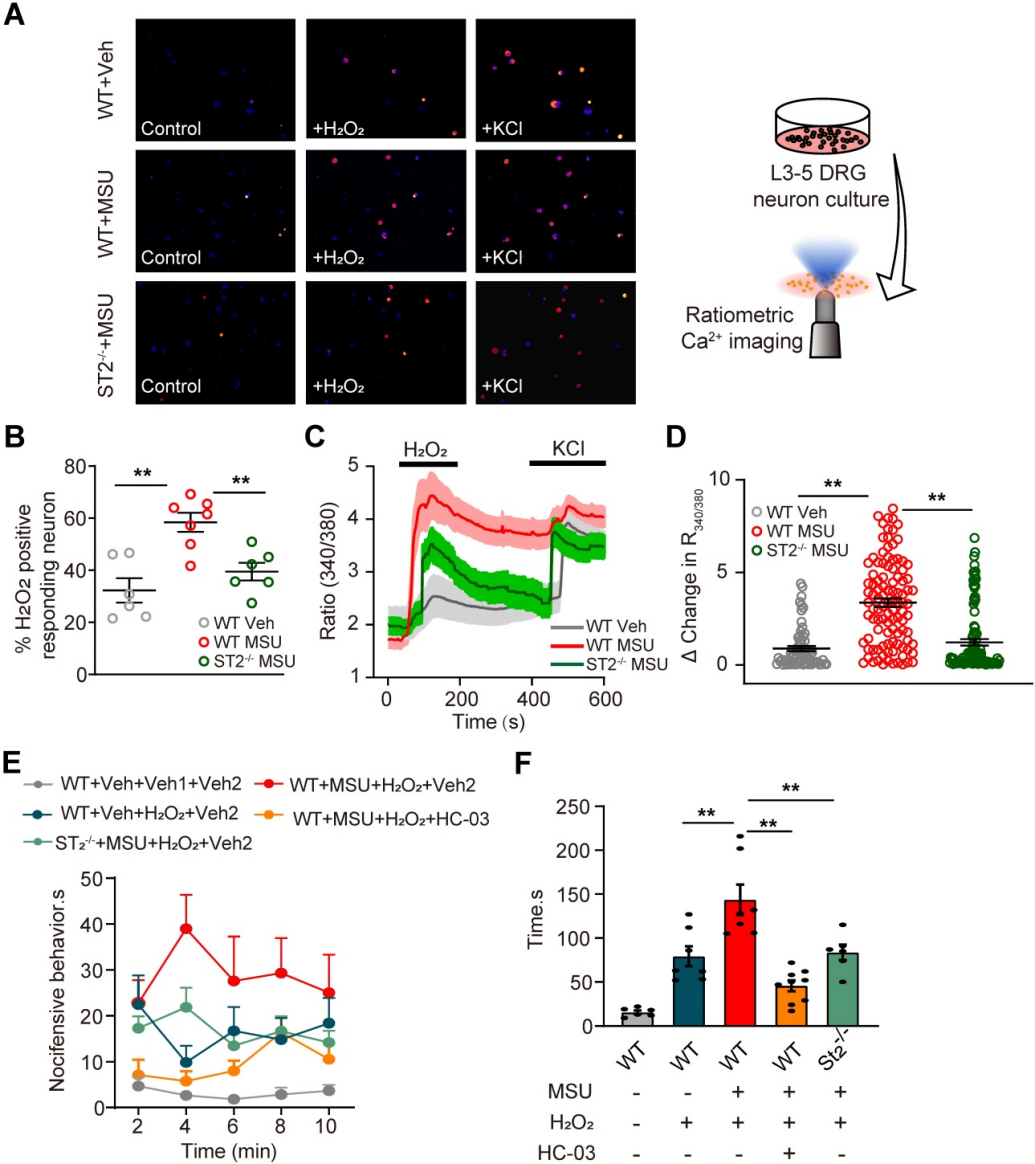
** TRPA1 channel activity is increased in DRG neurons of gout model mice via ST2 dependent manner, which enhances the nociceptive response to endogenous ROS products in gout condition.** (A) Pseudo color images from Fura-2-based ratiometric Ca^2+^ imaging indicating the Ca^2+^ responses from ipsilateral L3-5 DRG neurons in response to the endogenous TRPA1 agonist H_2_O_2_ (500 µM) in Veh (PBS) or MSU group mice. 40 mM KCl was perfused at the end of each recording to determine all live DRG neurons in observation field. (B) Summarized % of H_2_O_2_ positive responding DRG neurons in each observation field from Veh or MSU group of mice. n = 6-7 tests/group. Each group contains 150-200 neurons derived from 3-4 mice. (C) Comparison of averaged Ca^2+^ responses induced by 500 µM H_2_O_2_ between Veh and MSU group. Ca^2+^ traces are overlaid for comparison. n > 40 cells/group. (D) Summarized data showing Δ increase in peak 340/380 ratio before and after H_2_O_2_ application. n > 60 neurons/group derived from 3-4 mice/group. (E) Time course of the nocifensive behaviors of Veh and MSU group mice after H_2_O_2_/HC-030031 or corresponding vehicle injection. Veh: PBS, Veh1: PBS, Veh2: 1% DMSO (in PBS), HC-03: HC-030031. (F) Total time summary of all time points in panel (E). **p<0.01. One-way ANOVA followed by Tukey post hoc test was used for statistical analysis.

Endogenous ROS products, including H_2_O_2_ and 4-HNE, activate TRPA1 in peripheral sensory neurons to produce pain. Evidence suggests that MSU-induced gout arthritis increases TRPA1 expression in joint tissues 25. Thereby, we continued to explore whether TRPA1 channel activity was functionally enhanced in peripheral sensory neurons that innervate the ankles of MSU-treated mice. Ipsilateral L3-5 DRG neurons were acutely harvested from MSU- or PBS (Veh)-treated mice and loaded with Fura-2 for Ca^2+^ imaging (Figure 7A). The percentage of DRG neurons responding to endogenous TRPA1 agonist H_2_O_2_ (500 nM) was significantly higher in MSU group *vs*. Veh group (Figure 7A & B). In addition, the amplitude of Ca^2+^ responses elicited by H_2_O_2_ was significantly higher in MSU group *vs*. Veh group (Figure 7C & D). We also observed that the enhanced TRPA1 channel activity is reduced in DRG neurons from St2^-/-^ mice (Figure 7A-D). The *in vitro* results were further corroborated by *in vivo* behavioral tests. Injecting H_2_O_2_ (100 μg/site) into ipsilateral hind paws of MSU group mice resulted in more robust nocifensive behavior than Veh group mice (Figure 7E & F). The enhanced nocifensive response to H_2_O_2_ was eliminated by co-injecting mice with TRPA1 specific antagonist HC-030031 (10 μg/site, Figure 7E & F). Furthermore, H_2_O_2_ injection into MSU-treated ST2^-/-^ mice resulted in significantly reduced nociceptive response *vs*. MSU-treated WT mice (Figure 7E & F), a result consistent with the *in vitro* Ca^2+^ imaging results. Therefore, both* in vivo* and *in vitro* data suggests that TRPA1 channel activity is enhanced in DRG neurons of gout model mice via ST2 dependent mechanism, resulting in exaggerated nociceptive response to endogenous ROS products during gout.

## Discussion

In this study, we first screened the potential inflammatory cytokines or chemokines in the inflamed ankle tissue from a mouse gout arthritis model via RNA-Seq. We found that IL-33 was among the top up-regulated cytokines in the inflamed ankle. Neutralizing or genetic deleting IL-33 and its receptor ST2 significantly ameliorated pain hypersensitivities and inflammation in the mouse gout model. IL-33 promoted neutrophil influx and triggered neutrophil-dependent ROS production in the inflamed ankle, which activated TRPA1 channel in DRG neurons and produces nociception. Meanwhile, TRPA1 channel activity was significantly enhanced in DRG neurons that innervate the inflamed ankle, resulting in exaggerated nociceptive response to endogenous ROS products. These data all suggest a pivotal role of IL-33/ST2 in mediating gout pain and inflammation.

IL-33 can be released by several cell types, including keratinocytes, epithelial cells, fibroblasts and macrophages, etc. upon trauma, infection or inflammation 16, 31, 32. During gout, macrophages are the cells that accumulate in the inflamed tissues to react to MSU deposition by uptake of the crystals via phagocytosis 11. We found that both macrophage cell line RAW264.7 and cultured mouse peritoneal macrophages react to MSU challenge by producing IL-33* in vitro*. *In vivo* depletion of macrophages with clodronate significantly reduced IL-33 protein upregulation in ankle tissues of gout arthritis mice and further attenuated pain hypersensitivity and inflammation. This is by far the first evidence showing that macrophage reacts with MSU to produce IL-33. Therefore, our results suggest that macrophage may be among one of the cellular sources for IL-33 overproduction during gout. However, the participation of other cell types, such as fibroblasts, epithelial cells, etc. in IL-33 production under gout condition cannot be rule out and needs to be further tested.

Gout is accompanied with oxidative stress that provokes ROS production in local inflamed tissues 12. H_2_O_2_ has been found to be significantly increased in joint tissues from the mouse gout model and triggers hyperalgesia and inflammation via activating peripheral TRPA1 channel 13, 25. Our recent work identified that reducing ROS production by eucalyptol or ROS scavengers significantly reduced pain and inflammation in the mouse gout model, demonstrating an important role of ROS in mediating gout pain and inflammation 12. However, the exact mechanism of how oxidative stress is generated and modulated under gout condition is still not fully understood. In our study, we found that St2^-/-^ mice displayed significantly reduced oxidative stress, ROS production and pain hypersensitivity in gout condition, whereas exogenously injected IL-33 further promoted oxidative stress, ROS production and exacerbated pain hypersensitivity in gout condition via ST2 dependent mechanism. *In vivo* ROS imaging further indicated that ROS production in gout is related with ST2. These results in all suggest a pivotal role of IL-33/ST2 in modulating oxidative stress during gout.

Neutrophil influx into joint tissue is considered as the pathological hallmark of gout, which makes important contributions to gout pathogenesis 27. Here, we observed that St2^-/-^ mice showed much reduced neutrophil influx, whereas exogenously injected rIL-33 further promoted neutrophil influx into the inflamed ankle. This result suggests that IL-33/ST2 is involved in mediating neutrophil influx in gout. We found that St2^-/-^ mice showed much reduced tissue contents of CXCL1, CCL3 and IL-1β, which are neutrophil chemotactants crucial for neutrophil recruitment 33. This result indicates that CXCL1, CCL3 and IL-1β are produced via ST2 dependent mechanism in gout condition. IL-33 can act via ST2 expressed in macrophages to produce CXCL1, CCL3 and IL-1β during inflammation 33, 34. We reason here that IL-33, after being released from macrophages, may act in an autocrine manner to induce CXCL1, CCL3 and IL-1β production via ST2 in macrophages during gout. These cytokines or chemokines may then induce chemotaxis of neutrophils. Meanwhile, excellent previous studies have demonstrated in murine models and humans, IL-33 can directly promote neutrophil influx by acting on ST2 receptors expressed on neutrophils 33, 35. Thus, IL-33 may directly promote neutrophil influx by acting on ST2 expressed in neutrophils during gout via the paracrine mechanism. On the basis of these findings, we propose that IL-33 may act through ST2 via above mentioned autocrine or paracrine or both mechanisms to attract neutrophils in gout condition. This proposed mode of action is a reminiscent of IL-33-mediated neutrophil migration in rheumatoid arthritis, in which both autocrine and paracrine mechanisms as mentioned above are involved 33. Nonetheless, the exact cellular interactions that IL-33/ST2 mediated to promote neutrophil influx under gout condition remain to be further studied.

A recent study by Shang et al. reported that IL-33 attenuates inflammation in a MSU-induced mouse peritonitis model 15. They observed IL-33 treatment significantly reduced neutrophil influx in MSU-induced mouse peritonitis model. Moreover, IL-33 treatment reduced the inflammatory cytokine IL-1β and IL-6 production, while promoted anti-inflammatory cytokine IL-10, IL-5 and IL-13 production in peritoneal cavity gavage fluids. They further found that IL-33 recruited a subset of myeloid-derived suppressor cells, namely CD11b^+^Gr-1^int^F4/80^+^ cells, to suppress MSU-induced IL-1β production in peritoneal lavage fluids. These results are in contrast to our current observations. However, it is well established that IL-33 possesses unique biological function, which is about its seemingly contradictory pro-inflammatory *vs*. anti-inflammatory properties under different physical/pathological conditions 16. For example, in peritoneal cavity, IL-33 has been reported to promote the recruitment of IL-10-producing regulatory B cells to attenuate mucosal inflammatory responses 36. In contrast, in arthritis and some other conditions, IL-33 serves as a pro-inflammatory cytokine and exacerbates inflammation and pain via promoting the production of TNF-α, CXCL1, IL-1β and PGE_2_ through ST2 in plantar skin tissues 37-39. IL-33 also substantially potentiates substance P-induced TNF-α and IL-1β production from mast cells to enhance inflammation 40, 41. In addition, our recent work found that IL-33 released by keratinocytes can activate peripheral sensory neurons via promoting Ca^2+^ influx through TRPA1/TRPV1 channels in ST2 dependent mechanism, resulting in potentiation of skin inflammation and pruritus in a mouse allergic contact dermatitis model 20. The mouse gout model we used here is totally different from Shang et al., in which MSU was intra-articularly injected into the ankle of mice for one single time, whereas Shang et al. repetitively injected MSU into the peritoneal cavity for a total of 4 times during 4 days' period. It is believed that the biological effects of IL-33 is highly tissue specific and influenced by the microenvironment where it is produced 16. Given the fact that ankle cavity possesses totally different morphological structure, cell distributions and immune responses* vs*. peritoneal cavity, therefore, the different observations we had with Shang et al., may likely be attributed to different animal models we used. Nonetheless, the immunological mechanisms underlying the complex dichotomous functions of IL-33 still need to be further investigated.

TRPA1 is a target of endogenous ROS and lipid perioxidative products, such as OxPAPC, 4-HNE and H_2_O_2_ 42-45. TRPA1 is expressed by a subpopulation of peptidergic nociceptors and contributes to pain and neurogenic inflammation 43. Previous studies identified H_2_O_2_ level is significantly increased in the inflamed ankle tissue of gout model mice 25. Our study further identified that, in addition to H_2_O_2_, the lipid peroxidation product 4-HNE was also increased in the inflamed ankle tissues of gout model mice. It is reported that TRPA1 expression is upregulated in the inflamed joint tissues of gout model mice 25. We further demonstrated that TRPA1 channel activity is significantly enhanced in DRG neurons that innervate the inflamed ankle from gout model mice. This finding was further corroborated by the enhanced nocifensive response to the endogenous TRPA1 agonist H_2_O_2_ exhibited by gout model mice. We also observed that DRG neurons from St2^-/-^ mice showed much reduced TRPA1 activity upon H_2_O_2_ challenge. Certain inflammatory cytokines, such as IL-1β and IL-6 can promote TRPA1 expression in DRG neurons or chondrocytes 46, 47. IL-1β and IL-6 were both upregulated in the inflamed ankle tissue of gout model mice but much reduced in St2^-/-^ mice. Therefore, the enhanced TRPA1 channel functional activity we observed may be due to channel expression upregulation mediated by IL-1β, IL-6 or other inflammatory mediators released during gout.

## Conclusions

In all, our study demonstrated a previous unidentified role of IL-33/ST2 in mediating pain hypersensitivities and inflammation in a mouse gout model through promoting neutrophil-dependent ROS production and TRPA1 channel activation. Our study suggests that targeting IL-33/ST2 signaling may represent a novel therapeutic approach to ameliorate pain and inflammation in gout.

## Methods

### Animals

Male C57BL/6j or BALB/c mice (8-10 weeks; 20-25 g) were purchased from Shanghai Laboratory Animal Center, Chinese Academy of Sciences. St2 knockout (St2^-/-^) mice in the background of BALB/c were kindly provided by Dr. Andrew McKenzie at the MRC Laboratory of Molecular Biology, Cambridge, United Kingdom. Il-33 knockout (Il-33^-/-^) mice in the background of C57BL/6j were kindly provided by Dr. Hiroshi Kiyonari at the Laboratory for Animal Resources and Genetic Engineering, Center for Developmental Biology, Institute of Physical and Chemical Research, Kobe, Japan. All animals were housed in the Laboratory Animal Center of Zhejiang Chinese Medical University accredited by the Association for Assessment and Accreditation of Laboratory Animal Care (AAALAC) under standard environmental conditions (12 h light-dark cycle and 24 °C) with access to food and water provided *ad libitum*. The mice were randomly allocated and five mice were housed per cage. For group size estimation, we make sure that the data subjected to statistical analysis in our study has a group size (n) ≥ 5, according to our previous experience and studies using similar experimental protocols. We only included male mice in our study to circumvent hormonal female cycle possible interferences in behavioral analysis. Besides, gout has been considered as a male predominant disease and it occurs much more frequently in men than women 48.

### MSU-induced gout arthritis model establishment

Gout arthritis was induced by intra-articularly (i.a.) injection of MSU crystals (0.5 mg) suspended in 20 μl endotoxin-free phosphate buffered saline (PBS) into the tibio-tarsal joint (ankle) of mice under isoflurane anesthesia as we described before 12. Control group mice received an i.a. injection of 20 µL sterile PBS. A successful gout arthritis model establishment was judged by obvious swelling and mechanical hyperalgesia 2 h after MSU injection 13.

### Evaluation of ankle joint mechanical pain hypersensitivity

Mice were individually placed in transparent Plexiglas chambers on an elevated mesh floor and were habituated for 30 min before test. The mechanical hyperalgesia was determined using a series of von Frey filaments (0.04 to 4 g) applied perpendicularly to the heel area of the hind paw in ascending order beginning with the finest fiber. The minimum force that caused the mouse to withdraw its hind paw away from the filament was considered as the withdrawal threshold. For each mouse, a von Frey hair was applied 5 times at 10 s intervals. The threshold was determined when paw withdrawal was observed in more than three out of five applications as we previously described 49. The behavior tests are conducted by an experimenter blinded to experimental conditions.

### RNA-Seq and data processing

24 h post-injection of MSU or vehicle, mice were euthanized. The injected ankles were then collected, diced and stored in RNAlater (Thermal Fisher Scientific, Waltham, MA, USA). Total RNA from vehicle and MSU group was extracted using Trizol reagent (Thermal Fisher Scientific, Waltham, MA, USA). RNA quality and purity were validated by TapeStation (Agilent, Santa Clara, CA, USA) and NanoDrop (Thermo Fisher Scientific, Waltham, MA, USA). Only RNA samples showing RNA Integrity Number ≥8.0 and A260/230 ≥1.5 were used for RNA-Seq. The samples were sequenced by BGISEQ-500 from BGI Group (Shenzhen, China). Raw sequencing reads were aligned to mouse genome (mm10). Differential expression analyses were performed with R and Bioconductor packages of edgeR and limma voom as reported in our previous studies 50, 51. The threshold required for the genes to be considered significantly changed was as follows: q-value ≤0.001 and absolute value of |log_2_ (Fold Change)| ≥1.0.

### Myeloperoxidase (MPO) activity measurement

Neutrophil recruitment in ankle joint was evaluated via quantification of the enzyme MPO activity using a commercial MPO detection kit (Nanjing Jiancheng Bioengineering Institute, Nanjing, China) as we described 12. Briefly, mice were terminally anesthetized and the ankle joint was homogenized and centrifuged at 10, 000 rpm at 4 ℃ for 15 min. 10 µL of the supernatant was transferred into PBS (pH 6.0) containing 0.17 mg/ml 3, 3', 5, 5'-tetramethylbenzidine and 0.0005% H_2_O_2_. MPO catalyzed the redox reaction of H_2_O_2_ and 3, 3', 5, 5'-tetramethylbenzidine and produced yellow-colored compounds, through whose absorbance at 460 nm was determined. MPO activity was calculated and expressed as U/mg protein. One unit of MPO activity was defined as the quantity of enzyme that degraded 1 μmol H_2_O_2_ at 37 °C per g wet tissue.

### Air pouch model

Mice were anesthetized with isoflurane. The back skin was shaved and 3 ml of sterile air was subcutaneously injected (s.c.) into the back to establish air pouch as described 52. 3 days after the first injection, an additional 3 ml sterile air was injected into the pouch again. 3 days later, 3 mg of MSU crystals in 1 ml PBS, or 1 ml of PBS alone, was injected into the air pouches, respectively. 6 h after injection, mice were euthanized and exudates were collected and centrifuged at 500 g for 5 min at room temperature. Total cells were then counted with a hemocytometer and spun onto cytoslides and stained with Diff-Quick reagent. Differential cell counts were achieved by microscopic counting using standard morphological and staining criteria as we previously described 53.

### Cell culture

Raw 264.7 cells: Raw 264.7 macrophage cell line was purchased from Shanghai Academy of Life Sciences (Shanghai, China) and cultured in DMEM (Hycolon, USA) supplemented with 10% fetal bovine serum (FBS, BI, Israel), 100 units/mL penicillin and 100 μg/mL streptomycin at 37 °C in a humidified atmosphere of 5% CO_2_ and 95% air. DRG neurons: Mice were sacrificed 24 h after MSU injection. Ipsilateral L3-5 dorsal root ganglia (DRGs) were harvested and dissociated using collagenase type 1 and dispase (Gibco, Thermo Fisher Scintific, USA) as described previously 54, 55. DRG neurons were cultured in DMEM plus 10% FBS, 100 units/mL penicillin and 100 μg/mL streptomycin on round coverslips coated with poly-D-lysine (Sigma, MO, USA) and mouse laminin (Invitrogen, CA, USA) in 24-well chamber.

### Ca^2+^ imaging

DRG neurons were used 4 h after dissociation. Cells were loaded with Fura 2-AM (10 μM, Invitrogen) for 45 min in a loading buffer containing: NaCl 140, KCl 5, CaCl_2_ 2, MgCl_2_ 2, HEPES 10 (pH 7.4 adjusted with NaOH). Cells were subsequently washed 3 times and imaged in the loading buffer. Ratiometric Ca^2+^ imaging was performed on a Nikon ECLIPSE Ti-S (Japan) microscope with a Polychrome V monochromator (Till Photonics, USA) and an Orca Flash 4.0 CCD camera (Hamamatsu, Japan). Images were captured and processed with MetaFluor software (Molecular Devices, CA, USA). Ratiometric images were obtained with exposures of 0.5 ms at 340 nm and 0.3 ms at 380 nm excitation wavelengths. Representative Ca^2+^ imaging images were generated using ImageJ software. A cell was considered responsive if the peak Ca^2+^ response is above 20% of the baseline according to our previous publications 56, 57.

### *In vivo* ROS imaging

24 h after MSU injection into the right ankle, mice were injected (i.v.) with the fluorescent ROS indicator dye L-012 (Tocris, USA, 25 mg/kg), as described previously 58. Mice were anesthetized with isoflurane, then placed on the stage of an IVIS Lumina LT *in vivo* imaging system (PerkinElmer, Waltham, MA, USA) and the fluorescence imaging was performed 5 min post injection. The luminescence signal intensities were quantified with Living Image software (PerkinElmer, USA).

### Ethnic statement

This study was approved by the Laboratory Animal Management and Welfare Ethical Review Committee of Zhejiang Chinese Medical University (Permission Number: ZSLL-2017-183).

### Statistical analysis

Statistical analysis was conducted using SPSS 19.0 (IBM Corp., Armonk, NY, USA). Results were expressed as mean ± SEM. Student's t-test was used for comparisons between two groups. One-way or two-way ANOVA followed by Tukey's post hoc test was used for comparison among groups ≥ 3. Comparison is considered significantly different if p < 0.05.

## Supplementary Material

Supplementary figures and tables.

## Figures and Tables

**Table 1:** The expression (pg/mg protein) of some inflammatory cytokines or chemokines in ankle tissues of wild type and ST2^-/-^ mice treated with vehicle (Veh, PBS) or MSU, measured by Bio-Plex multiplex immunoassays. n=5 mice/group

Cytokines	WT+Veh	WT+MSU	St2^-/-^+Veh	St2^-/-^+MSU
IL-1α	220.0±6.2	275.6±12.7	191.4±8.16	189.8±28.3^##^
IL-1β	1.9±0.2	4.2±0.2**	1.1±0.3	2.9±0.2^##^
IL-2	3.1±0.4	4.3±0.2**	2.7±0.2	3.4±0.2
IL-5	0.9±0.1	1.3±0.1**	0.7±0.1	1.3±0.1
IL-6	1.3±0.2	7.9±0.8**	1.3±0.3	4.9±0.2^##^
IL-10	8.9±0.9	9.8±0.3	9.5±0.9	9.1±0.7
IL-12	14.3±0.9	20.6±1.4**	11.5±0.6	18.8±0.8
IL-13	9.8±1.4	24.9±1.3**	9.1±2.7	16.9±0.4^#^
CCL11	48.3±3.6	116.5±15.6**	48.9±4.2	52.0±3.7^##^
G-CSF	6.2±1.3	30.7±2.8**	6.9±1.6	19.8±1.3^##^
CXCL1	17.4±2.4	76.7±7.7**	13.7±2.2	27.8±3.0^##^
CCL2	228.6±51.3	1423.5±85.5**	202.5±91.6	598.1±47.1^##^
CCL3	16.9±3.6	83.8±9.2**	12.5±3.4	39.3±2.8^##^
CCL5	154.7±30.2	287.0±76.8**	113.8±27.3	169.4±34.7

**p<0.01 *vs*. WT+Veh, ^#^p<0.05, ^##^p<0.01 *vs*. WT+MSU. One-way ANOVA followed by Tukey`s post hoc test was used for statistical analysis.
