# Supplementary material for: IL-33/ST2 induces neutrophil-dependent reactive oxygen species production and mediates gout pain
Source: Theranostics. 2020 Oct 27;10(26):12189–203. doi: 10.7150/thno.48028 (PMC7667675; doi:10.7150/thno.48028)
Supplement: Supplementary file 1 — Supplementary figures and tables. [file thnov10p12189s1.pdf]

Supplementary Materials

Title page

Title
IL-33/ST2 induces neutrophil-dependent reactive oxygen species production and mediates gout pain

Author

Chengyu Yin[1, #], Boyu Liu[1, #], Yuanyuan Li[1], Xiaojie Li[1], Jie Wang[1], Ruixiang Chen[1], Yan Tai[2], Qiyang Shou[3], Ping Wang[4], Xiaomei Shao[1], Yi Liang[1], Hong Zhou[5], Wenli Mi[6], Jianqiao Fang[1,*] and Boyi Liu[1,*]

# These authors contributed equally to this work.

*Correspondence: Boyi Liu, Department of Neurobiology and Acupuncture Research, The Third Clinical Medical College, Zhejiang Chinese Medical University, Key Laboratory of Acupuncture and Neurology of Zhejiang Province, Hangzhou, 310053, China. E-mail: boyi.liu@zcmu.edu.cn

Or Jianqiao Fang, Department of Neurobiology and Acupuncture Research, The Third Clinical Medical College, Zhejiang Chinese Medical University, Key Laboratory of Acupuncture and Neurology of Zhejiang Province, Hangzhou, 310053, China. E-mail: fangjianqiao7532@163.com.

Abstract

Objective: Gout, induced by monosodium urate (MSU) crystal deposition in joint tissues, provokes severe pain and impacts life quality of patients. However, the mechanisms underlying gout pain are still incompletely understood. **Methods:** We established a mouse gout model by intra-articularly injection of MSU crystals into the ankle joint of wild type and gentic knockout mice. RNA-Sequencing, *in vivo* molecular imaging, Ca^{2+} imaging, reactive oxygen

species (ROS) generation, neutrophil influx and nocifensive behavioral assay, etc. were used. **Results:** We found interleukin-33 (IL-33) was among the top up-regulated cytokines in the inflamed ankle. Neutralizing or genetic deletion of IL-33 or its receptor ST2 (suppression of tumorigenity) significantly ameliorated pain hypersensitivities and inflammation. Mechanistically, IL-33 was largely released from infiltrated macrophages in inflamed ankle upon MSU stimulation. IL-33 promoted neutrophil influx and triggered neutrophil-dependent ROS production via ST2 during gout, which in turn, activated transient receptor potential ankyrin 1 (TRPA1) channel in dorsal root ganglion (DRG) neurons and produced nociception. Further, TRPA1 channel activity was significantly enhanced in DRG neurons that innervate the inflamed ankle via ST2 dependent mechanism, which results in exaggerated nociceptive response to endogenous ROS products during gout. **Conclusions:** We therefore demonstrated a previous unidentified role of IL-33/ST2 in mediating pain hypersensitivity and inflammation in a mouse gout model through promoting neutrophil-dependent ROS production and TRPA1 channel activation. Targeting IL-33/ST2 may represent a novel therapeutic approach to ameliorate gout pain and inflammation.

Keywords: Gout; arthritis; TRPA1; reactive oxygen species; cytokine; neutrophil;

Graphical abstract

IL-33 produced by macrophages upon MSU stimulation promoted neutrophil influx and triggered neutrophil-dependent ROS and lipid peroxidation products generation via ST2, which activated TRPA1 in DRG neurons and produced gout pain.

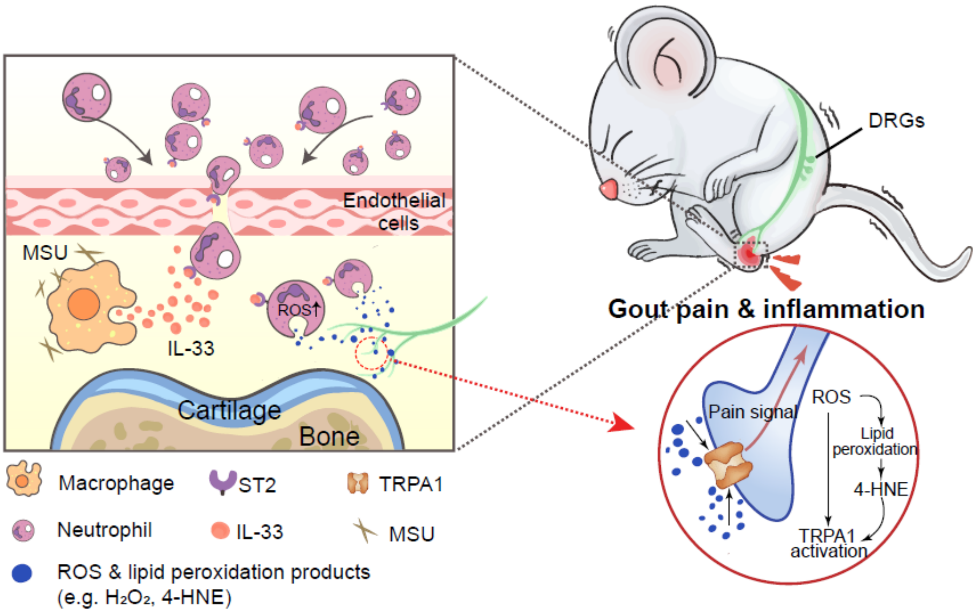

Supplementary methods and materials

Chemicals

MSU and DMSO were purchased from Sigma (St. Louis. MO, USA). H_2O_2 and LPS were purchased form Solarbio Life Sciences (Beijing, China). Fucoidin was purchased from APExBIO Technology (Houston, TX, USA). Clodronate and liposome controls were purchased from Liposoma BV (Amsterdam, The Netherlands). Fura-2 AM was purchased from Abcam (Cambridge, UK). HC-030031 and L-012 were purchased from Tocris (Minneapolis, MN, USA).

Ankle edema test

Ankle edema was evaluated as an % increase in ankle diameter measured with a digital caliper and was calculated as follows as previously described [1, 2]: % increase in ankle diameter=$(L_{after}-L_{basal})/L_{basal} \times 100\%$. The experimenter was blind to experimental conditions. The same experimenter carried out all edema tests to avoid inter-observer variability.

H_2O_2-induced nocifensive behavior assay

Mice were placed into transparent chambers and habituated for 30 minutes before testing. Chemicals were injected into the hind paw of mice using 1-mL syringe and 30-gauge needle as follows: H_2O_2 (100 μg/site) was co-injected with HC-030031 (10 μg/site) or vehicle in a total volume of 10 μl dissolved in PBS. Nocifensive behavior (licking, flinching, or biting of injected paw) was recorded with a videorecorder for 10 min and quantified thereafter. All behavior tests are conducted by an experimenter blinded to experimental conditions.

The open field test

The test consists of a non-transparent Plexiglass enclosure (40 cm × 40 cm × 30 cm) placed in a sound-proof experimental room. Animals were habituated to experimental room conditions for 30 min before test. To initiate testing, the mice were placed in the center of the field individually, and the total distance they traveled in the field was monitored by the camera above the field during 5 min testing time. The movement was then analyzed by Anymaze software from Stoelting (Wood Dale, IL, USA) and compared thereafter.

ELISA test

Ankles joint samples were harvested after treatment, weighed and immediately frozen in liquid nitrogen. Tissues were homogenized with Bullet Blender (BBX24, NextAdvance Inc. NY, USA) in 50 mM Tris-base (PH 7.4) and 150 mM NaCl added with protease inhibitors (Roche, Switzerland) and 0.2% Triton X-100 (Sigma, MO, USA) at 4 ℃ for 20 min at full speed as we described previously. The supernatants were centrifuged at 12, 000g for 12 min at 4 ℃. The protein concentrations were determined by BCA assay (Thermo Fisher Scientific, Waltham, MA, USA). The supernatants were tested by ELISA in duplicates for IL-33 using a commercial kit (R&D Systems, Minneapolis, MN, USA), according to the manufacture's instruction.

Bio-Plex multiplex immunoassays

The concentrations of some key inflammatory cytokines or chemokines in supernatant of homogenized ankle tissues of mice were detected using Bio-Plex multiplex immunoassay (RayBiotech, Norcross, GA, USA). Briefly, a total of 50 μl antibody-conjugated beads were added

to the assay plate. The samples were diluted in 1:24. 50 μl of diluted samples, standards, the blank, and controls were added to the plate, respectively. The plate was then incubated in the dark with shaking at 850 rpm for 30 min and then washed 3 times with 100 μl washing buffer. A total of 25 μl biotinylated antibody and 50 μl streptavidin-phycoerythrin were added to the plate, respectively, with shaking and washing procedure mentioned above. The readout was carried out using Bio-Plex protein array reader and Bio-Plex Manager 6.0 was used for data acquisition and analysis.

Thioglycollate-elicited macrophages (TPMs) culture

TPMs were generated by injecting the mice (i.p.) with 2.5 ml of 4 % Brewer's thioglycollate medium (Sigma, USA) as described before [3]. Mice were euthanized 72 h after injection. Peritoneal cavity cells were harvested by lavage, and cells were washed and plated in 6-well chamber. Cells were cultured in DMEM plus 10% FBS, 100 units/mL penicillin and 100 μg/mL streptomycin. Cells were incubated overnight and adherent cells were then stimulated with MSU or LPS.

Macrophage depletion with clodronate

200 μl of a clodronate-laden liposome suspension (5 mg/ml) was injected (i.p.) 48 h before behavioral test. Liposomes lacking clodronate served as vehicle control. The efficiency of macrophage depletion was evaluated by immunostaining the spleen and periarticular ankle tissues with F4/80 antibody.

Neutrophil aggregation blocking with fucoidine

Mice were treatment with fucoidin (20 mg/kg in 20 μl) or corresponding vehicle (PBS) by intravenous injection via the tail vein at 8 h and 23.5 h after MSU injection. The efficiency of neutrophil aggregation blockage was evaluated by MPO assay.

IL-33/ST2 neutralizing antibody application

For blocking IL-33 and ST2 signaling, mouse IL-33–neutralizing and ST2-neutralizing antibodies (catalog numbers: AF3626 and MAB10041; R&D Systems) and normal goat or rat isotype control IgGs (catalog numbers: I5256 and I4131; Sigma-Aldrich) were dissolved in sterile PBS. For blocking IL-33, IL-33 neutralizing antibody (10 μg/mouse) was treated at 0 h, 8 h and 23 h time points, for a total of three times, after MSU injection. In cases where ST2-neutralizing antibody was co-applied with rIL-33, ST2 neutralizing antibody (50 μg/mouse) was pre-mixed with rIL-33 (300 ng/mouse) and co-injected with rIL-33 into the ankle joint of mice. The effective dosages of IL-33- and ST2-neutralizing antibodies were derived from our previous study [4].

Determination of oxidant/anti-oxidant status

Ankle joint tissues from mice of each group were collected 24 h after MSU injection. The samples were then homogenized, followed by centrifugation at 3,000 rpm for 15 min. Supernatant was collected for superoxide dismutase (SOD), reduced glutathione (GSH) and malondialdehyde (MDA) assay as we described before [1]:

Detection of SOD activity: SOD activity was detected based on the inhibition of nitro blue tetrazolium reduction using the xanthine/xanthine oxidase system as a superoxide generator with a

commercially available kit (Nanjing Jiancheng Bioengineering Institute, Nanjing, China). Absorbance was determined with a microplate reader at 560 nm. The results were averaged and expressed as U/mg of sample protein.

Detection of MDA level: Lipid peroxidation product MDA level was determined based upon the concentration of thiobarbituric acid-reactive substances using a commercially available kit (Beyotime, Shanghai, China). Absorbance was determined using a microplate reader at 532 nm. MDA concentration was calculated using the absorbance coefficient according to manufacturer's instruction and expressed as U/mg of sample protein.

Detection of GSH-Px level: GSH-Px level was measured using 5, 5′-dithiobis-(2-nitrobenzoic acid) recycling method with a commercially available kit (Nanjing Jiancheng Bioengineering Institute, Nanjing, China). Absorbance was monitored using a microplate reader at 412 nm. The results were deduced from comparison with that of the standard solution of GSH and expressed as μmol/mg of sample protein.

Immunofluorescent staining

Mice were deeply anesthetized with isoflurane and were perfused through the ascending aorta with 0.9% saline followed by 4% paraformaldehyde in 0.1 M PBS. After perfusion, the spleen and periarticular tissues were removed and post-fixed in the same fixative for 4-6 h (4 ℃) before transferring to 30% sucrose for 72 h for dehydration. Tissues were serially cut into 8 μm thickness section on a frozen microtome (CryoStar NX50, Thermo Fisher, CA, USA) and processed for immunofluorescence. The sections were first blocked with 5% donkey serum in PBS (with 0.3% Triton X-100, blocking buffer) for 1 h at 37 ℃ and then incubated overnight at 4 ℃ with the

following primary antibodies diluted in blocking buffer: rat anti F4/80 (1:500, Abcam). The sections were then incubated 1 hr at 37 °C with mixture of corresponding secondary antibodies. Fluorescence images were captured by Nikon A1R laser scanning confocal microscope. The sections were examined and analyzed in a blinded manner. 3 images were randomly selected per mouse tissue and averaged and compared as described in our previous study [5].

Western blotting

After measuring protein concentrations, the samples were heated for 5 min with 5X loading buffer at 100 °C and loaded onto SDS-PAGE. The proteins were electrophoretically transferred onto polyvinylidene fluoride membranes. After being blocked with 5% nofatty milk in Tris-buffer saline containing 0.1% Tween-20 for 1h at room temperature, the membranes were then probed with following primary antibodies overnight at 4 °C. These antibodies included goat anti-IL33 (1:500, R&D Systems), goat anti-ST2 (1:1000, R&D Systems), rabbit anti-4HNE (1:500, Abcam) and mouse anti-β-actin (1:5000, Abcam). The proteins were then detected by horseradish peroxidase-conjugated and corresponding secondary antibodies (1:5000, CST). The results of protein expression are normalized to the density of β-actin. Fold change in control/vehicle group was expressed as 100% quantification.

Real-time quantitative PCR (qPCR)

At 24 h after MSU injection, the mice were euthanized and ankle joint samples were collected. Total RNA from each group was extracted in 1 mL Trizol reagent and centrifuged at 12,000 rpm at 4 °C for 10 min. The extracted total RNA from ankle was reverse-transcribed into cDNA using

random haxamers primers with Prime ScriptTM RT reagent Kit (TaKaRa Bio Inc, China). Each reaction was performed in triplicates and normalized to β-actin gene expression. qPCR was performed in CFX96 Instrument Sequence Detection System (Bio-Rad, Berkeley, CA, USA) using Fast Start Universal SYBR Green Master Kit (TaKaRa Bio Inc, China) with 25 μL reaction system. The CT value of each well was determined using the CFX96 Real-Time System Software and the average of the triplicates was calculated. The relative quantification was determined by the △△CT method as described before [6]. The sequences of all primers used were shown in Table S1.

Supplementary figures and legends:

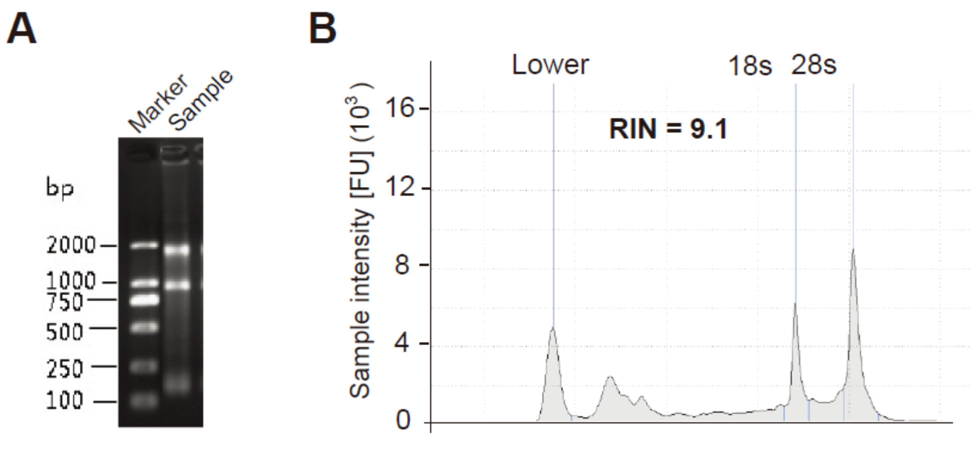

Figure S1 High quality ankle tissue RNA obtained for RNA-Seq. (A) Ankle tissue RNA electrophoresis image. Left lane shows the marker and right lane shows the RNA sample. (B) RNA integrity number (RIN) determined by TapeStation.

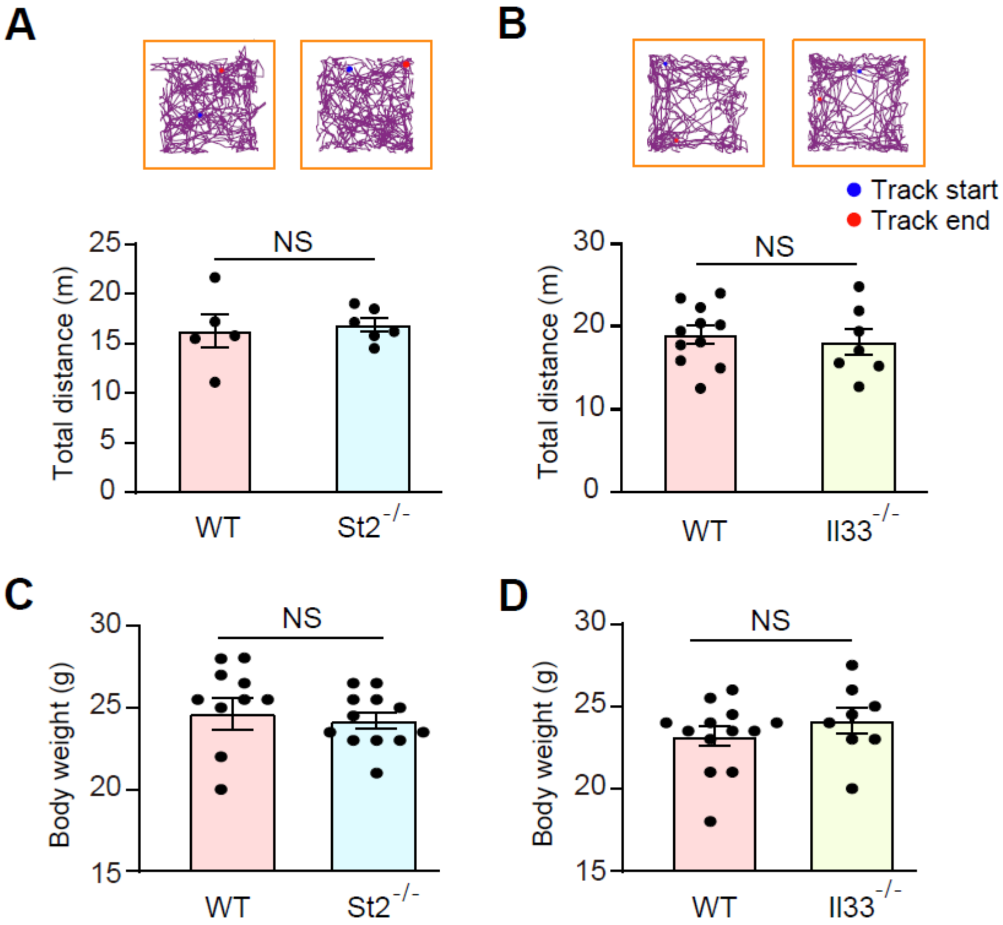

Figure S2 St2$^{-/-}$ and Il33$^{-/-}$ mice showed normal general locomotor activities in open field test and body weight. (A&B) Open field test of naïve St2$^{-/-}$ (A) and Il33$^{-/-}$ (B) mice compared with WT control mice. Upper panel: typical movement traces of the mice. Lower panel: Summarized total distance the mice traveled. (C&D) Comparison of body weight of St2$^{-/-}$ (C) or Il33$^{-/-}$ (D) mice with WT control mice. NS: no significance. Student's *t* test is used for statistical analysis.

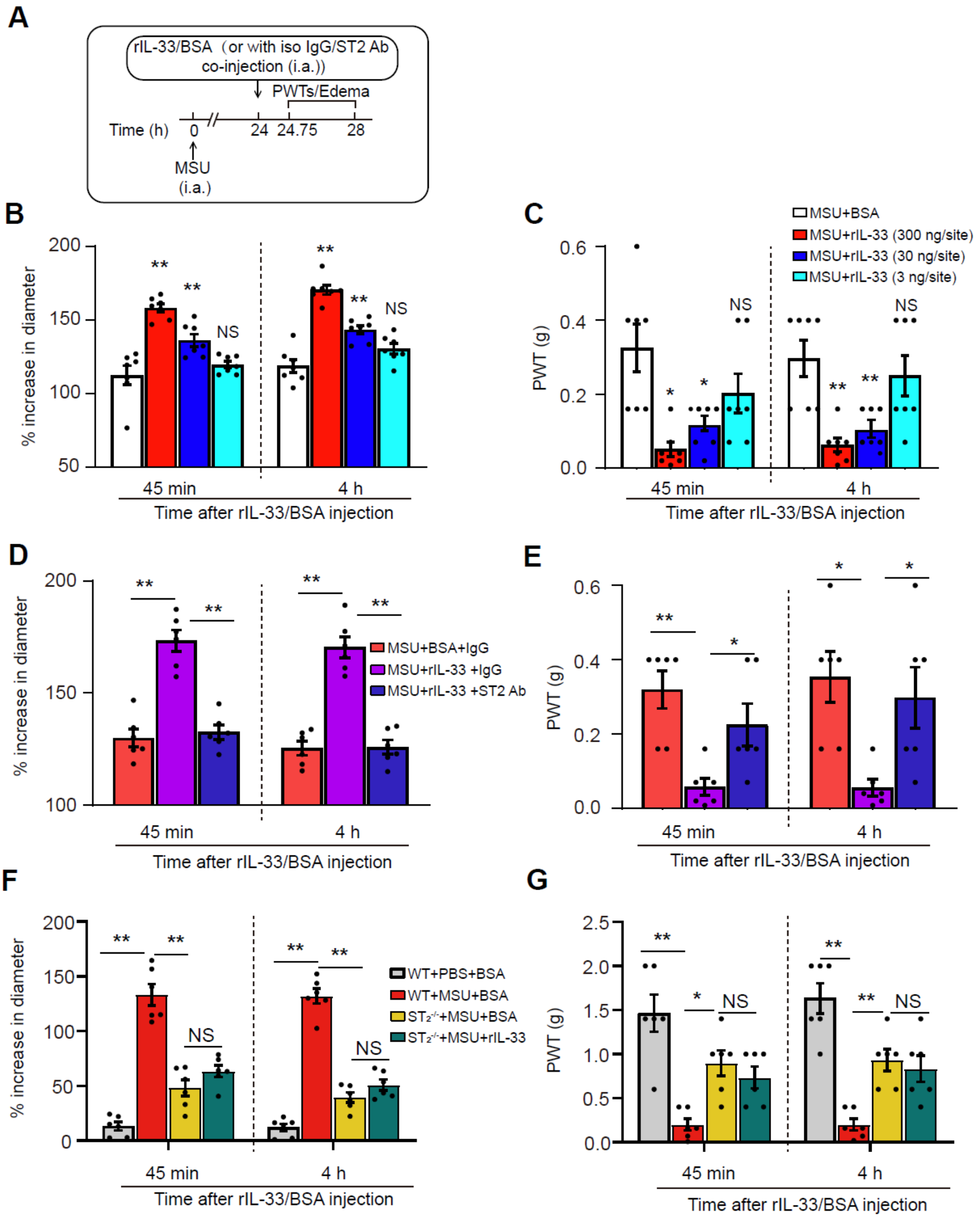

Figure S3 Exogenously injected recombinant IL-33 exacerbates pain hypersensitivities and ankle edema of gout model mice via ST2 dependent mechanism. (A) Schematic protocol for gout model establishment and time points for i.a. injection of rIL-33/BSA (or in combination with ST2 neutralizing antibody/Iso-type IgG). Ankle diameter and PWTs were observed 45 min and 4 h after rIL-33/BSA injection. (B) % increase in ankle diameter after 3, 30 or 300 ng rIL-33 or BSA injection. (C) PWT changes after 3, 30 or 300 ng rIL-33 or BSA injection. (D) % increase in ankle diameter after IL-33 (300 ng) combined with ST2 neutralizing antibody (50 µg) or iso-type IgG injection. (E) PWT changes after IL-33 (300 ng) combined with ST2 neutralizing antibody (50 µg) or iso-type IgG injection. (F) % increase in ankle diameter after IL-33 or BSA injection into WT or $St2^{-/-}$ mice. (G) PWT changes after IL-33 or BSA injection into WT or $St2^{-/-}$ mice. n = 5-7 mice/group. $^{*}p<0.05$ and $^{**}p<0.01$. NS: no significance. One-way ANOVA followed by Tukey post hoc test was used for statistical analysis.

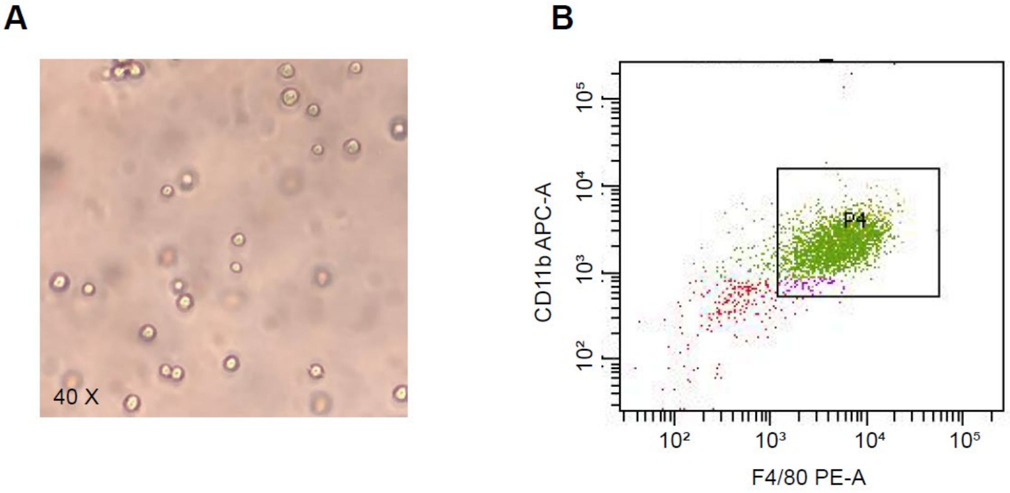

Figure S4 Thioglycollate-elicited macrophage collection and identification. (A) TPMs observed under transmitted light microscope. (B) FACS plots showing the percentage of F4/80$^+$/CD11b$^+$ macrophages in peritoneal fluids after thioglycollate treatment.

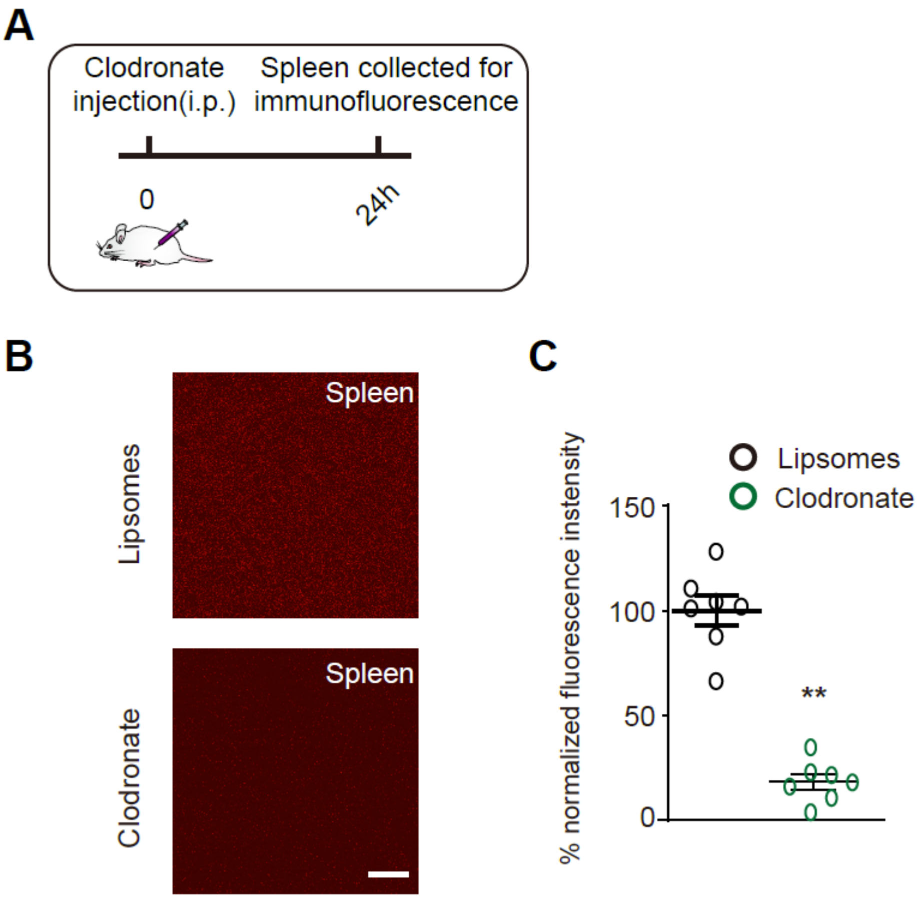

Figure S5 Validation of clodronate-induced macrophage depletion. (A) Schematic protocol for the time points of clodronate or liposome application and spleen tissue collection. (B) Representative immunofluroscence images showing spleen section stained with macrophage marker F4/80 in clodronate or liposome-treated group. (C) Summarized % normalized fluorescence intensity of (B). n = 7 mice/group. Scale bar indicate 50 µm. **p<0.01. Student's *t* test is used for statistical analysis.

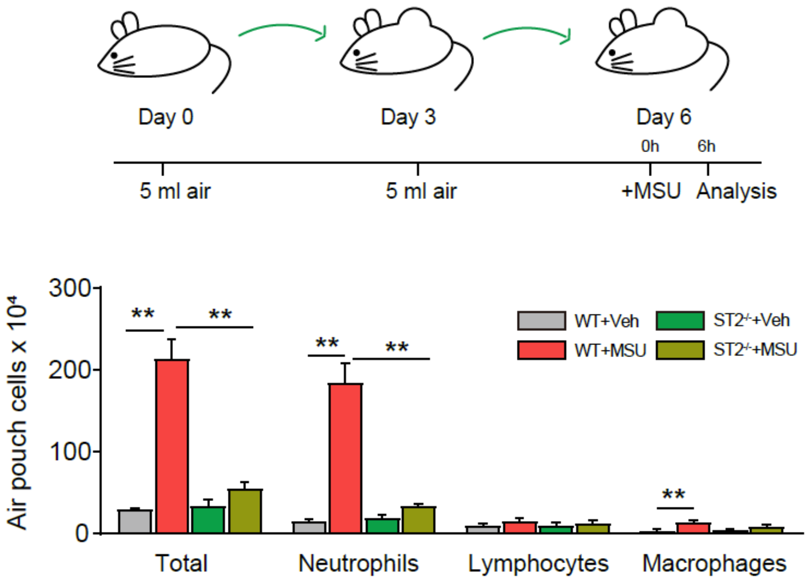

Figure S6 Analysis of infiltrated cells in tissue exudates from the mouse air pouch model. Upper panel: Protocol for establishing the mouse air pouch model. On Day 0 and 3, 5 ml sterile air was injected subcutaneously (s.c.) into the back of the mouse. On Day 6, MSU (3 mg/ml, in 1 ml injection volume) or PBS (1 ml) was injected into the air pouches. 6 h later, the animals were sacrificed and tissue exudates were collected for analysis. Lower panel: Cell counts (total, neutrophils, lymphocytes and macrophages) from tissue exudates of the air pouch model from WT and $St2^{-/-}$ mice. n = 5-6 mice/group. **p<0.01. One-way ANOVA followed by Tukey post hoc test was used for statistical analysis.

Table S1. Primers sequence used for qPCR evaluation of gene expression changes in the present study

Gene name	Gene ID	Primer sequence (5′ to 3′)	Amplicon size (bp)
β-actin	11461	F:5’-GTGCTATGTTGCTCTAGACTTCG-3’	174
		R:5’-ATGCCACAGGATTCCATACC-3’	
Il-33	77125	F:5’-CAGAAGACCAAAGAATTCTGCC-3’	130
		R:5’-CATGCTTGGTACCCGATTTTAG-3’	
St2	17082	F:5’-TGACACCTTACAAAACCCGGA-3’	178
		R:5’-AGGTCTCTCCCATAAATGCACA-3’	
Il-1rap	16180	F:5’-CACCAAGGTACACAGTAGAACT-3’	123
		R:5’-CAGCCAGTAAACATGGTAAACC-3’	

References

1. Yin C, Liu B, Wang P, Li X, Li Y, Zheng X, et al. Eucalyptol alleviates inflammation and pain responses in a mouse model of gout arthritis. Br J Pharmacol. 2020; 177: 2042-57.
2. Silva CR, Oliveira SM, Hoffmeister C, Funck V, Guerra GP, Trevisan G, et al. The role of kinin B1 receptor and the effect of angiotensin I-converting enzyme inhibition on acute gout attacks in rodents. Ann Rheum Dis. 2016; 75: 260-8.
3. Hannemann N, Cao S, Eriksson D, Schnelzer A, Jordan J, Eberhardt M, et al. Transcription factor Fra-1 targets arginase-1 to enhance macrophage-mediated inflammation in arthritis. J Clin Invest. 2019; 129: 2669-84.
4. Liu B, Tai Y, Achanta S, Kaelberer MM, Caceres AI, Shao X, et al. IL-33/ST2 signaling excites sensory neurons and mediates itch response in a mouse model of poison ivy contact allergy. Proc Natl Acad Sci U S A. 2016; 113: E7572-E9.
5. Hu Q, Zheng X, Li X, Liu B, Yin C, Li Y, et al. Electroacupuncture Alleviates Mechanical Allodynia in a Rat Model of Complex Regional Pain Syndrome Type-I via Suppressing Spinal CXCL12/CXCR4 Signaling. J Pain. 2020.
6. Livak KJ, Schmittgen TD. Analysis of relative gene expression data using real-time quantitative PCR and the 2(-Delta Delta C(T)) Method. Methods. 2001; 25: 402-8.
